# Supplementary material for: Behavioral role of PACAP signaling reflects its selective distribution in glutamatergic and GABAergic neuronal subpopulations
Source: eLife. 2021 Jan 19;10:e61718. doi: 10.7554/eLife.61718 (PMC7875564; doi:10.7554/eLife.61718)
Supplement: Figure 1—source data 1. — Adcyap1, PACAP-mRNA mapping within glutamatergic and GABAergic subpopulations of mouse brain. Slc17a7, Slc17a6, and Slc32a1: mRNAs encoding the VGLUT1, VGLUT2, and VGAT with respective color coding for the chromogens labeling the corresponding mRNAs. Double arrows indicate examples of colocalization of two probe mRNAs and single arrows indicate no-co-expression in given cells. Panels A–O: coronal and sagittal sections taken from the indicated Bregma or medio-lateral coordinates. The combination of mRNA probes is indicated with respective colors corresponding to the DISH method end products. The high-magnification panels are examples with relevant molecular features, of the regions labeled within low-magnification photomicrographs (see the abbreviations vide infra). Note that the high-magnifications photos are not from the same experiment of the low magnification. 3 v: third ventricle; ACA: anterior cingulate area; ac: anterior commissure; AHN: anterior hypothalamic nucleus; AI: agranular insular area; AONpv: anterior olfactory nucleus, postero-ventral; AUD: auditory areas; AVPV: antero-ventral periventricular nucleus; BAC: bed nucleus of anterior commissure; BSTpl: Bed nucleus of the stria terminalis, posterolateral; CA3v: ventral hippocampal formation, CA3; CBpj: purkinje layer of the cerebellum; CENT: central lobule of the cerebellum; COA: cortical amygdala; CSm: superior central nucleus raphe, medial part; CSl: superior central nucleus raphe, lateral part; DCO: dorsal cochlear nucleus; DR: Dorsal raphe nucleus; DMHa: dorsomedial nucleus of the hypothalamus, anterior part; ECT: ectorrinal area; ENT: entorrinal area; FL: flocculus; NLOT: nucleus of the lateral olfactory tract; gcl: granule cell layer; GU: gustatory area; IC: inferior colliculus; ICe: inferior colliculus external nucleus; IF: interfascicular nucleus of the raphe; IL: infralimbic area; IPN: interpeduncular nucleus; ISN: inferior salivatory nucleus; KF: Koelliker-Fuse subnucleus; LH: lateral habenula; [file elife-61718-fig1-data1.pdf]

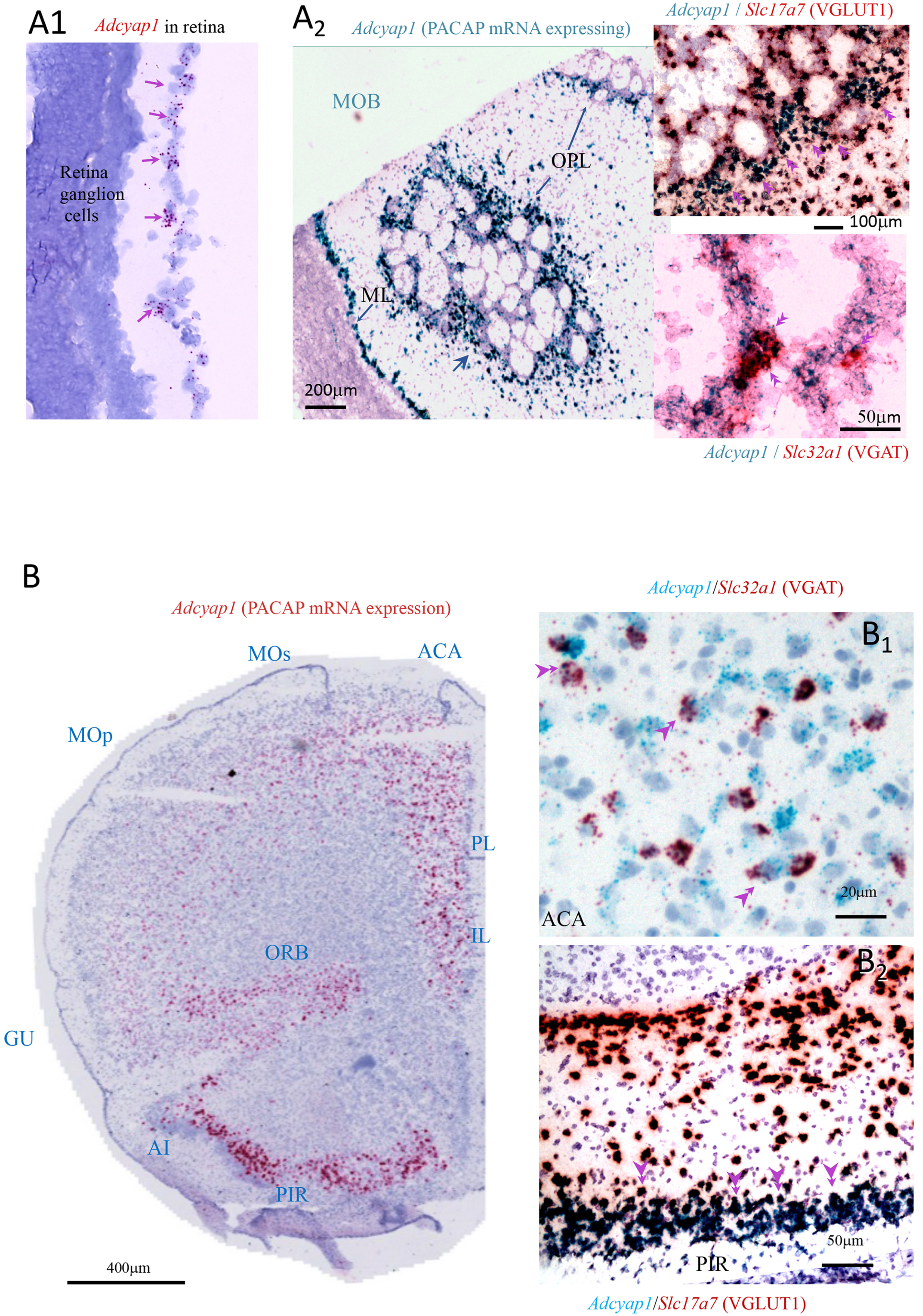

A1
Adcyap1 in retina
Retina ganglion cells
A2
Adcyap1 (PACAP mRNA expressing)
MOB
OPL
ML
200µm
Adcyap1 / Slc17a7 (VGLUT1)
100µm
50µm
Adcyap1 / Slc32a1 (VGAT)
B
Adcyap1 (PACAP mRNA expression)
MOs
ACA
MOp
PL
IL
ORB
GU
AI
PIR
400µm
Adcyap1/Slc32a1 (VGAT)
B1
ACA
20µm
B2
PIR
50µm
Adcyap1/Slc17a7 (VGLUT1)

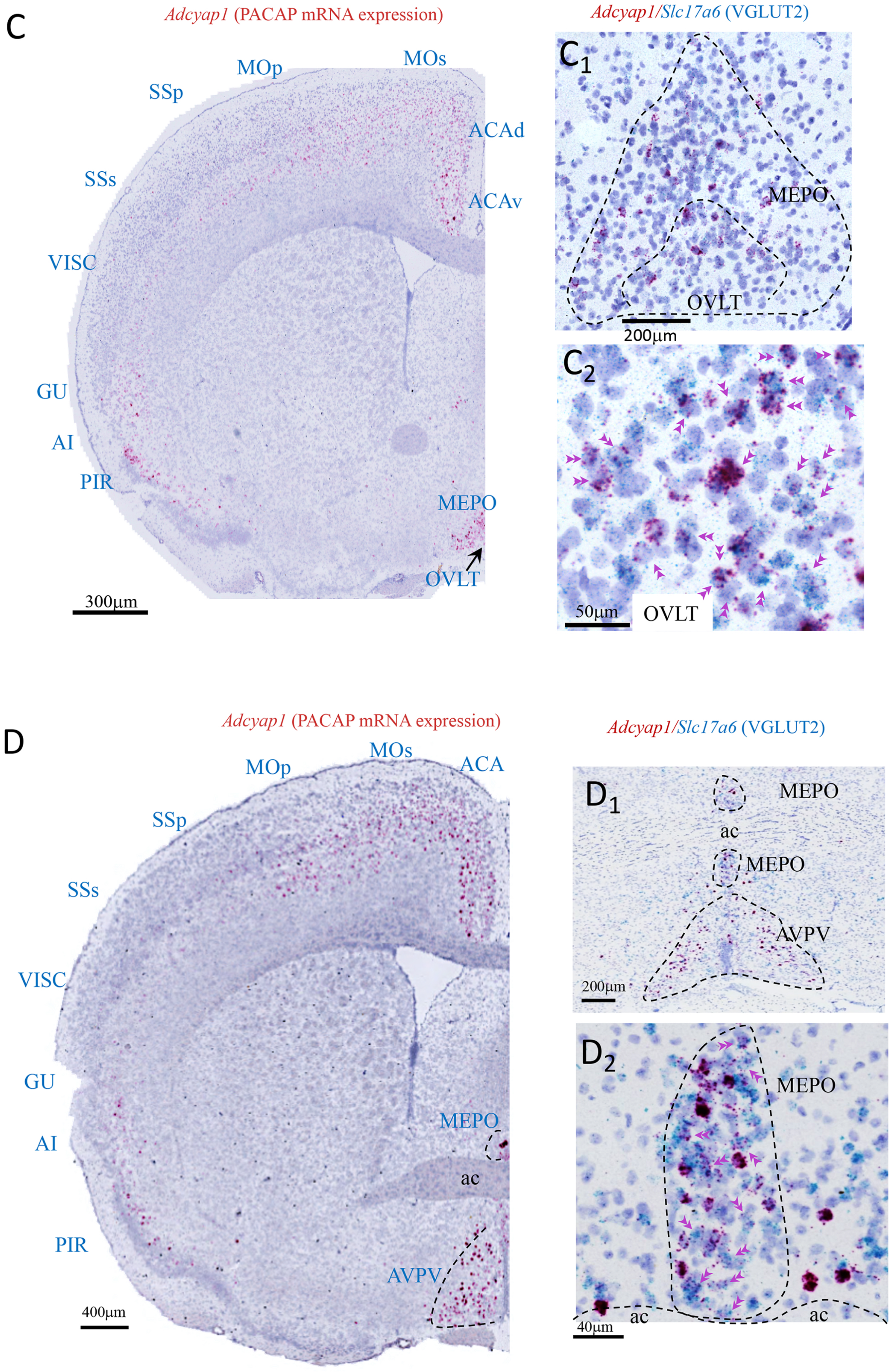
C
*Adcyap1* (PACAP mRNA expression)
MOp
MOs
SSp
ACAd
SSs
ACAv
VISC
GU
AI
PIR
MEPO
OVLT
300µm
*Adcyap1*/*Slc17a6* (VGLUT2)
C1
MEPO
OVLT
200µm
C2
50µm
OVLT
D
*Adcyap1* (PACAP mRNA expression)
MOs
ACA
MOp
SSp
SSs
VISC
GU
AI
MEPO
ac
PIR
AVPV
400µm
*Adcyap1*/*Slc17a6* (VGLUT2)
D1
MEPO
ac
MEPO
AVPV
200µm
D2
MEPO
ac
ac
40µm

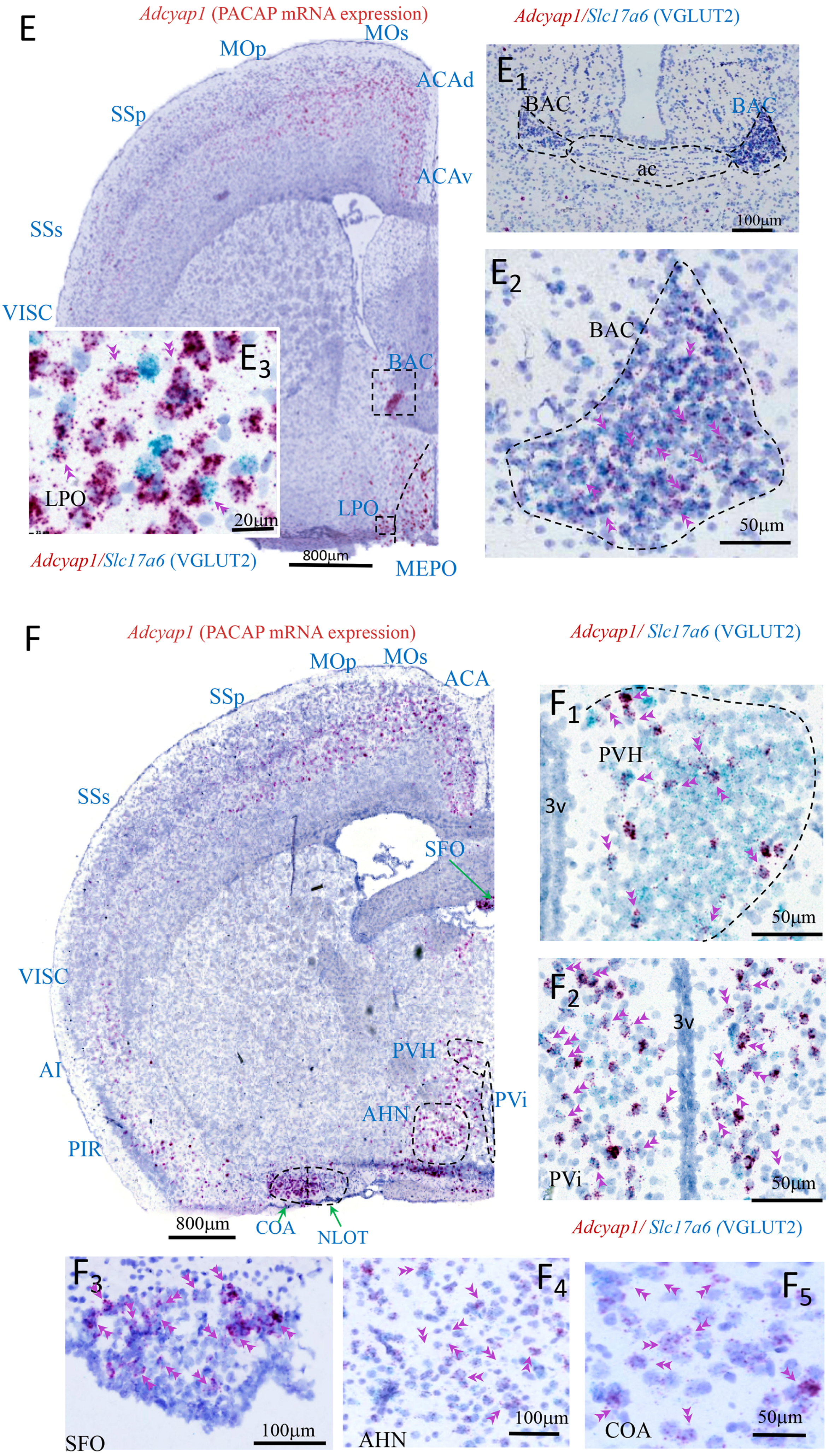

E
Adcyap1 (PACAP mRNA expression)
MOp
MOs
ACAd
SSp
ACAv
SSs
VISC
BAC
LPO
MEPO
800µm
E3
LPO
20µm
Adcyap1/Slc17a6 (VGLUT2)
Adcyap1/Slc17a6 (VGLUT2)
E1
BAC
BAC
ac
100µm
E2
BAC
50µm
F
Adcyap1 (PACAP mRNA expression)
MOp
MOs
ACA
SSp
SSs
SFO
VISC
AI
PIR
PVH
PVi
AHN
COA
NLOT
800µm
Adcyap1/ Slc17a6 (VGLUT2)
F1
PVH
3v
50µm
F2
3v
PVi
50µm
Adcyap1/ Slc17a6 (VGLUT2)
F3
SFO
100µm
F4
AHN
100µm
F5
COA
50µm

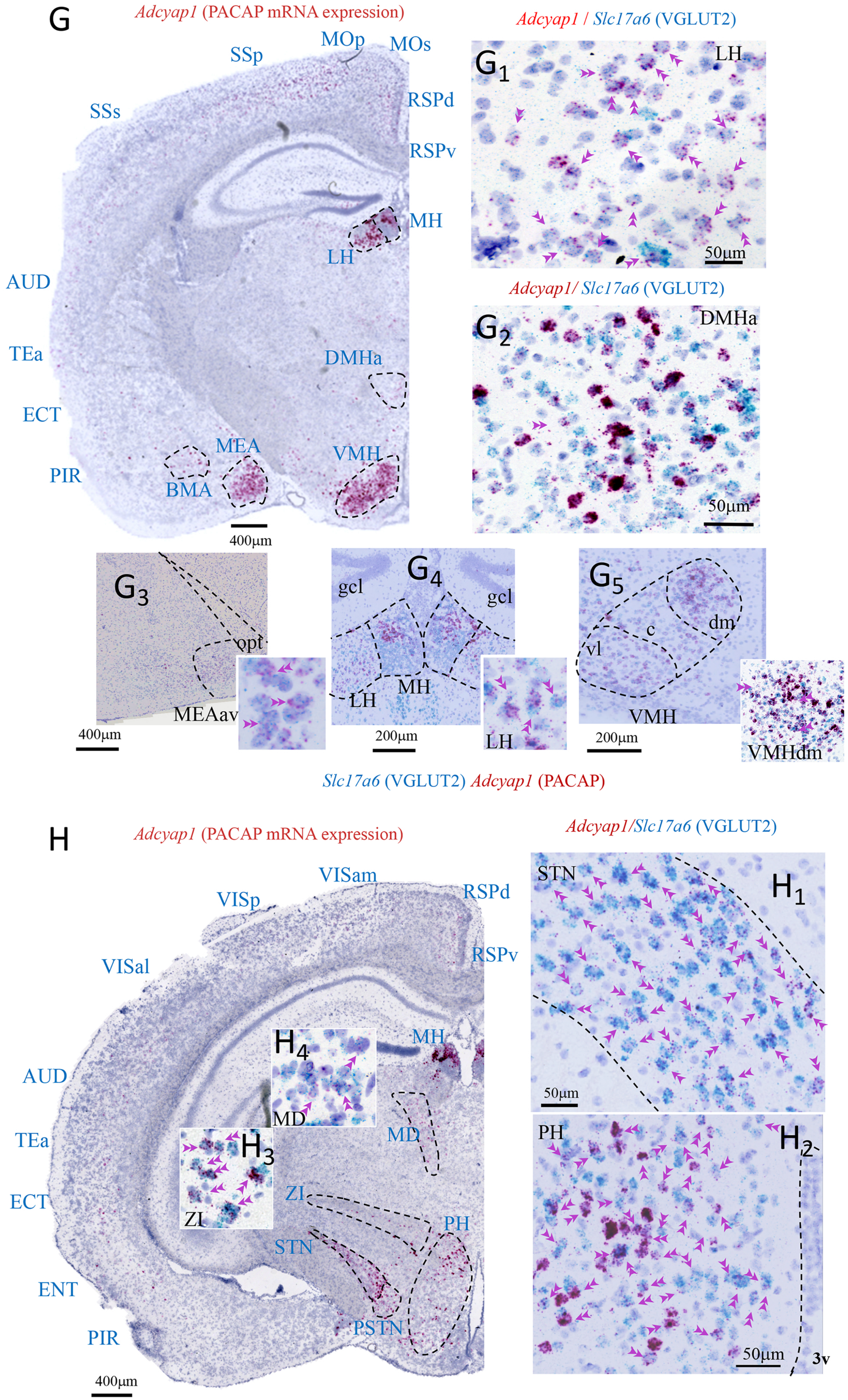
G
Adcyap1 (PACAP mRNA expression)
SSp
MOp
MOs
SSs
RSPd
RSPv
MH
LH
AUD
TEa
DMHa
ECT
MEA
VMH
PIR
BMA
400µm
Adcyap1 / Slc17a6 (VGLUT2)
G1
LH
50µm
Adcyap1/ Slc17a6 (VGLUT2)
G2
DMHa
50µm
G3
opt
MEAav
400µm
G4
gcl
gcl
MH
LH
200µm
LH
G5
dm
c
vl
VMH
200µm
VMHdm
Slc17a6 (VGLUT2) Adcyap1 (PACAP)
H
Adcyap1 (PACAP mRNA expression)
VISam
VISp
RSPd
VISal
RSPv
MH
H4
MD
AUD
MD
TEa
H3
ZL
ECT
ZI
PH
STN
ENT
PSTN
PIR
400µm
Adcyap1/Slc17a6 (VGLUT2)
STN
H1
50µm
PH
H2
50µm
3v

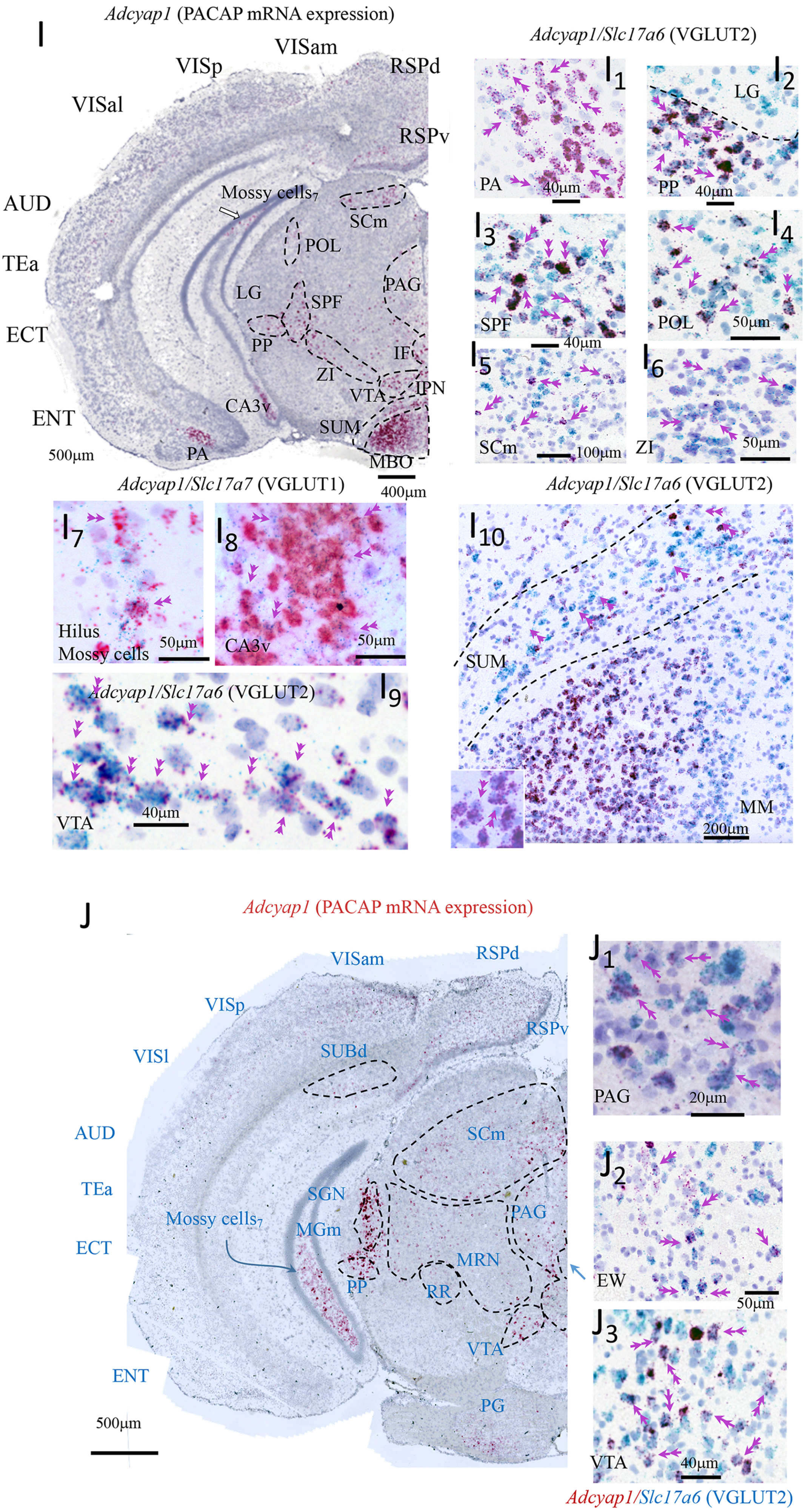
I
Adcyap1 (PACAP mRNA expression)
VISp
VISam
RSPd
VISal
RSPv
AUD
TEa
ECT
ENT
Mossy cells7
SCm
POL
LG
SPF
PAG
PP
IF
ZI
VTA
IPN
CA3v
SUM
PA
MBO
500µm
400µm
Adcyap1/Slc17a6 (VGLUT2)
I1
PA
40µm
I2
LG
PP
40µm
I3
SPF
40µm
I4
POL
50µm
I5
SCm
100µm
I6
ZI
50µm
Adcyap1/Slc17a7 (VGLUT1)
I7
Hilus
Mossy cells
50µm
I8
CA3v
50µm
Adcyap1/Slc17a6 (VGLUT2)
I9
VTA
40µm
Adcyap1/Slc17a6 (VGLUT2)
I10
SUM
MM
200µm
J
Adcyap1 (PACAP mRNA expression)
VISam
RSPd
VISp
RSPv
VISl
SUBd
AUD
SCm
TEa
SGN
PAG
Mossy cells7
MGm
ECT
MRN
PP
RR
VTA
ENT
PG
500µm
J1
PAG
20µm
J2
EW
50µm
J3
VTA
40µm
Adcyap1/Slc17a6 (VGLUT2)

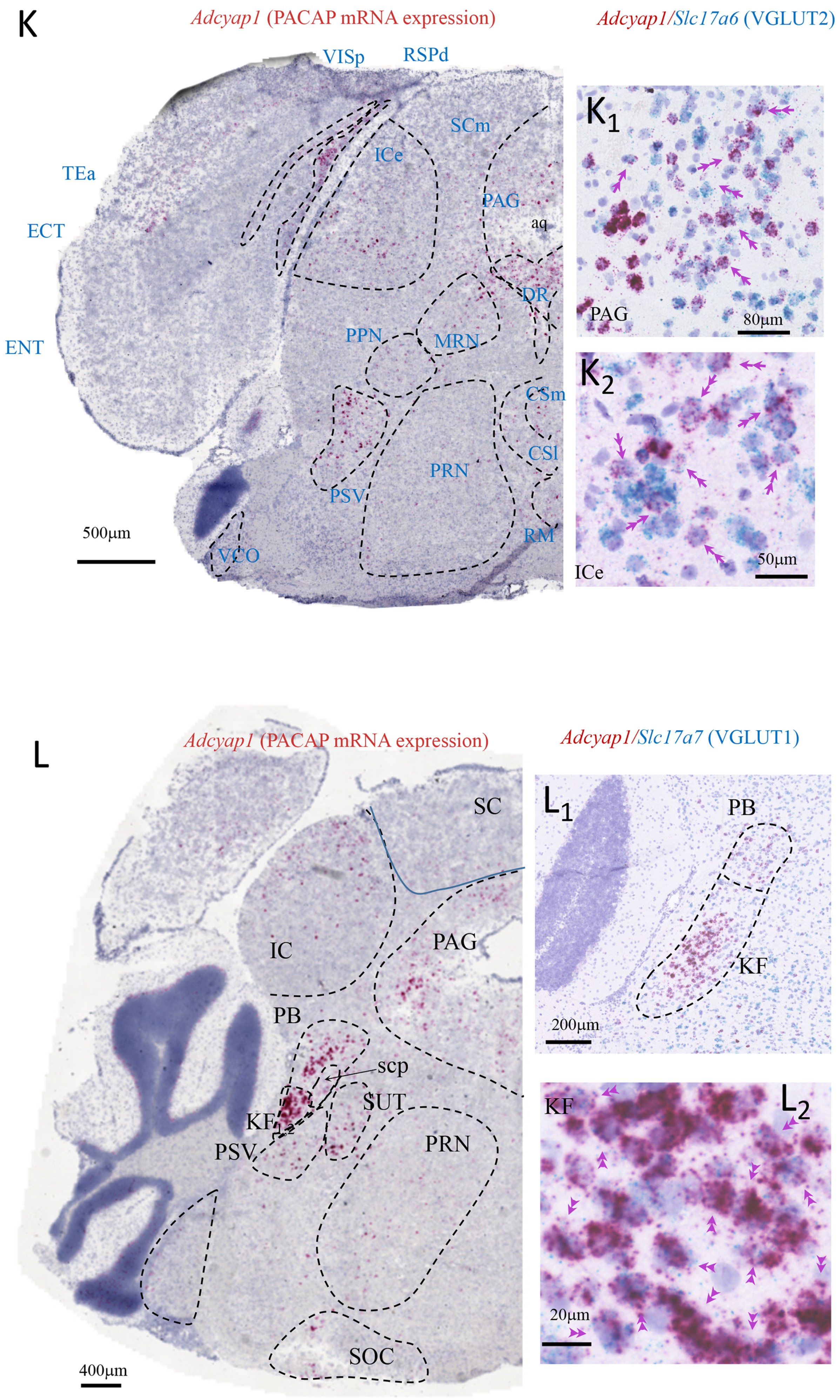
K
Adcyap1 (PACAP mRNA expression)
Adcyap1/Slc17a6 (VGLUT2)
VISp
RSPd
SCm
ICe
TEa
ECT
ENT
PAG
aq
DR
PPN
MRN
CSm
CSl
PRN
PSV
RM
VCO
500µm
K1
PAG
80µm
K2
ICe
50µm
L
Adcyap1 (PACAP mRNA expression)
Adcyap1/Slc17a7 (VGLUT1)
SC
IC
PAG
PB
scp
SUT
KF
PSV
PRN
SOC
400µm
L1
PB
KF
200µm
L2
KF
20µm

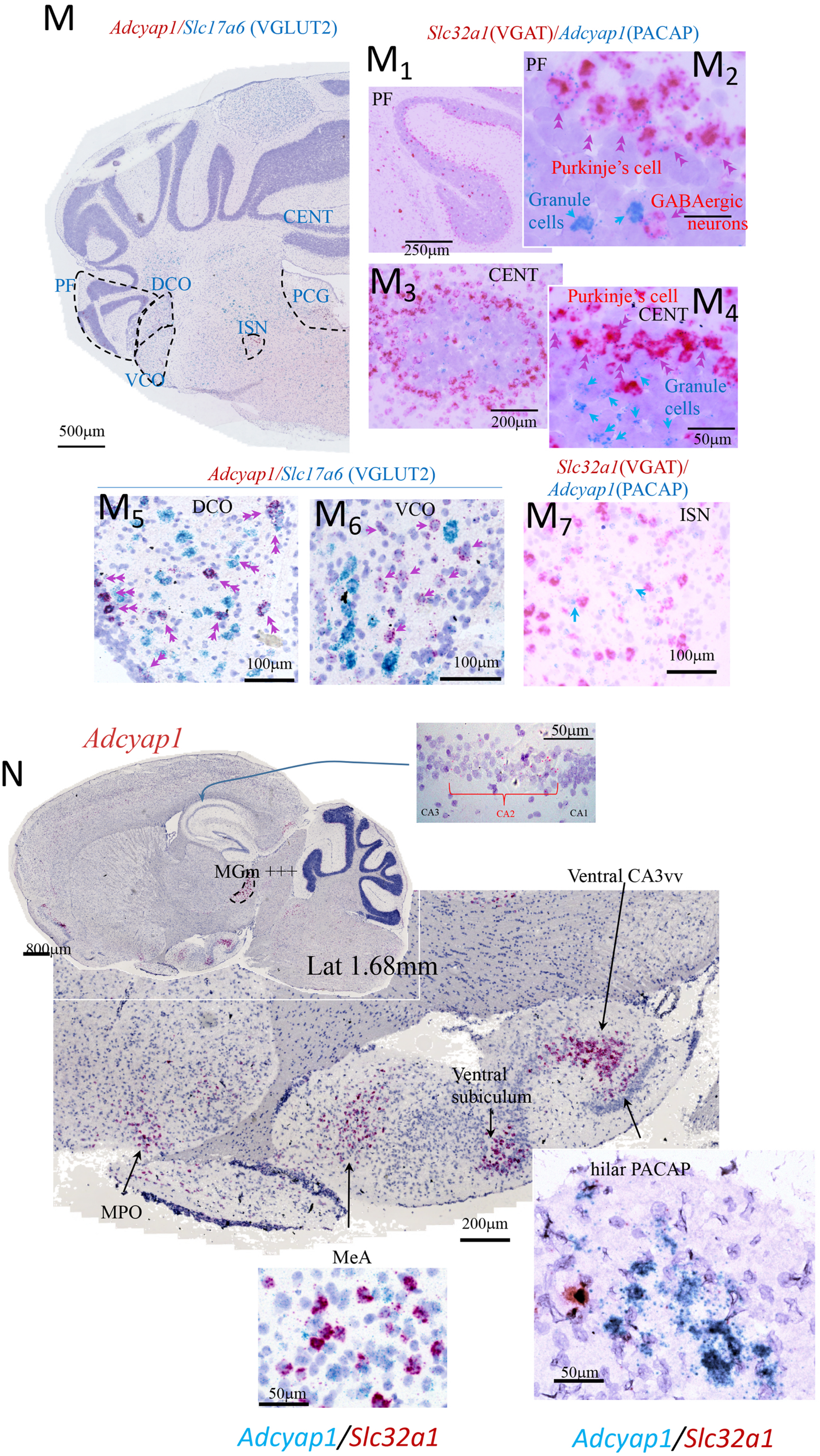
M
Adcyap1/Slc17a6 (VGLUT2)
CENT
PF
DCO
PCG
ISN
VCO
500µm
Slc32a1(VGAT)/Adcyap1(PACAP)
M1
PF
250µm
M2
PF
Purkinje's cell
Granule cells
GABAergic neurons
M3
CENT
200µm
M4
Purkinje's cell
CENT
Granule cells
50µm
Adcyap1/Slc17a6 (VGLUT2)
M5
DCO
100µm
M6
VCO
100µm
Slc32a1(VGAT)/Adcyap1(PACAP)
M7
ISN
100µm
Adcyap1
N
50µm
CA3
CA2
CA1
MGm +++
800µm
Lat 1.68mm
Ventral CA3vv
Ventral subiculum
hilar PACAP
MPO
200µm
MeA
50µm
50µm
Adcyap1/Slc32a1
Adcyap1/Slc32a1

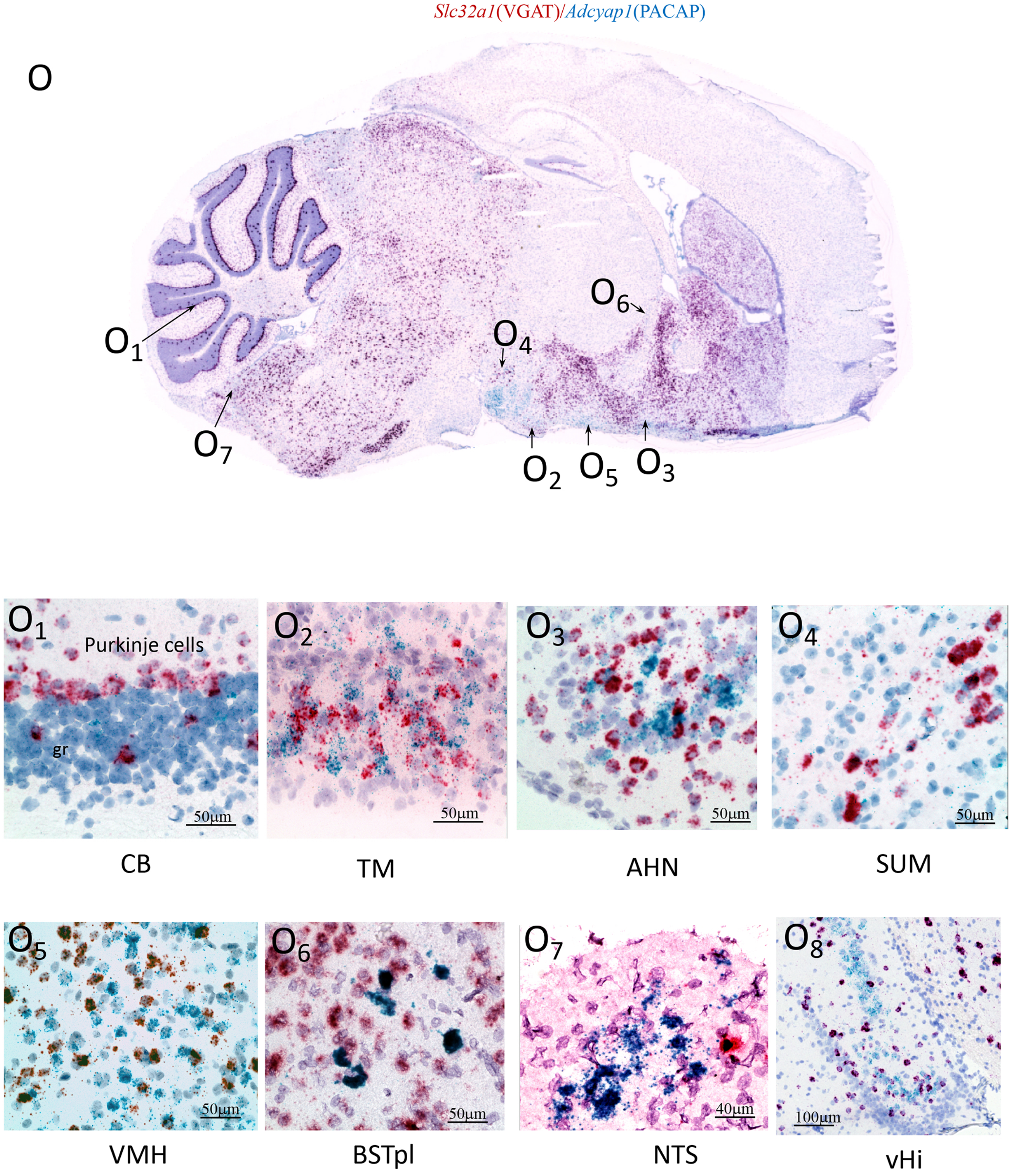

Slc32a1(VGAT)/Adcyap1(PACAP)
O
O1
O2
O3
O4
O5
O6
O7
Purkinje cells
gr
50µm
40µm
100µm
CB
TM
AHN
SUM
VMH
BSTpl
NTS
vHi
O8

**Figure 1 - source data**

**Comprehensive DISH mapping of PACAP co-expression with VGLUT1, VGLUT2 and VGAT throughout mouse brain reveals an extensive distribution and diversity of cell types.** *Adcyap1*, PACAP-mRNA mapping within glutamatergic and GABAergic subpopulations of mouse brain. *Slc17a7*, *Slc17a6* and *Slc32a1*: mRNAs encoding the VGLUT1, VGLUT2 and VGAT, with respective color coding for the chromogens labeling the corresponding mRNAs. Double arrows indicate examples of colocalization of two probe mRNAs and single arrows indicate no-co-expression in given cells. Panels **A-O:** coronal and sagittal sections taken from the indicated Bregma or medio-lateral coordinates. The combination of mRNA probes is indicated with respective colors corresponding to the DISH method end products. The high magnification panels are examples with relevant molecular features, of the regions labeled within low magnification photomicrographs (see the abbreviations *vide infra*). Note that the high magnifications photos are not from the same experiment of the low magnification. Abbreviations: 3v: third ventricle; ACA: anterior cingulate area; ac: anterior commissure; AHN: anterior hypothalamic nucleus; AI: agranular insular area; AONpv: anterior olfactory nucleus, postero-ventral; AUD: auditory areas; AVPV: antero-ventral periventricular nucleus; BAC: bed nucleus of anterior commissure; BSTpl: Bed nucleus of the stria terminalis, posterolateral; CA3v: ventral hippocampal formation, CA3; CBpj: purkinje layer of the cerebellum; CENT: central lobule of the cerebellum; COA: cortical amygdala; CSm: superior central nucleus raphe, medial part; CSl: superior central nucleus raphe, lateral part; DCO: dorsal cochlear nucleus; DR: Dorsal raphe nucleus; DMHa: dorsomedial nucleus of the hypothalamus, anterior part; ECT: ectorrinal area; ENT: entorrinal area; FL: flocculus; NLOT: nucleus of the lateral olfactory tract; gcl: granule cell layer; GU: gustatory area; IC: inferior colliculus; ICe: inferior colliculus external nucleus; IF: interfascicular nucleus of the raphe; IL: infralimbic area; IPN: interpeduncular nucleus; ISN: inferior salivatory nucleus; KF: Koelliker-Fuse subnucleus; LH: lateral habenula; ISN: inferior salivatory nucleus; LPO: lateral preoptic area; MD: mediodorsal nucleus of the thalamus; MEA: medial amygdala; MEPO: median preoptic nucleus; MGm: medial geniculate complex, medial part; MH: medial habenula; MM: medial mammillary nucleus; MOB: main olfactory bulb; MOp: primary motor area; MOs: supplemental motor area; MPO: medial preoptic area; MRN: midbrain reticular nucleus; NTS: nucleus of the tractus solitarius; opt: optic tract; ORB: orbital area; OV: vascular organ of lamina terminalis; PA: posterior amygdalar nucleus; PAG: periaqueductal gray; PB: parabrachial nucleus; PCG: contine central gray; PG: pontine gray; PH: posterior hypothalamic nucleus; PIR: piriform area; PL: prelimbic area; POL: posterior limiting nucleus of the thalamus; PP: peripeduncular nucleus; PPN: pedunculo pontine nucleus; PRN: pontine reticular nucleus; PSTN: parasubthalamic nucleus; PSV: principal sensory nucleus of the trigeminal nerve; RR: midbrain reticular nucleus, retrorubral area; RSPd: retrosplenial area dorsal; RSPv: retrosplenial area dorsal; PVH: periventricular hypothalamic nucleus; PVi: periventricular hypothalamic nucleus, intermediate part; RM: raphe magnus; SC: superior colliculus; SCm: superior colliculus, motor related; SFO: subfornical organ; SPF: subparafascicular nucleus; STN: subthalamic nucleus; SUBd: subiculum dorsal part; SGN: suprageniculate nucleus; SOC: superior olivary complex; SUM: supramammillary nucleus; TEa: Temporal association area; TM : tuberomammillary nucleus; VCO: ventral coclear nucleus; VISam: anteromedial visual área; VISp: primary visual area; VISal: anterolateral visual area; VISl: lateral visual area; VISC: visceral area; vHi: ventral hippocampus; VMH: ventromedial hypothalamic nucleus; VMHc: ventromedial hypothalamic nucleus, central part; VMHdm: ventromedial hypothalamic nucleus, dorsomedial part; VMHvl: ventromedial hypothalamic nucleus, ventrolateral part; VTA: ventral tegmental area; ZI: zona incerta.
