# Supplementary material for: Behavioral role of PACAP signaling reflects its selective distribution in glutamatergic and GABAergic neuronal subpopulations
Source: eLife. 2021 Jan 19;10:e61718. doi: 10.7554/eLife.61718 (PMC7875564; doi:10.7554/eLife.61718)
Supplement: Figure 3—source data 1. — Each panel from A to J show a low-magnification image of the coronal section analyzed, indicating with arrows the regions where the corresponding high-magnification photomicrographs were taken. The red signal corresponds to SLC32a1 (the mRNA for VGAT) and the green signal correspond to Vipr1 (the mRNA for VPAC1). The abbreviatures correspond to the Allen Brain Map and are indicated in the Figure 3—source data 5, where a comparison with the expression observed in the Vipr1 ISH experiments from Allen (73927619 and 77924538) and a semiquantitative analysis of the co-expression with VGAT mRNA was done. Scale bar: 2 mm for low-amplification and 50 µm for high-amplification photomicrographs. [file elife-61718-fig3-data1.pdf]

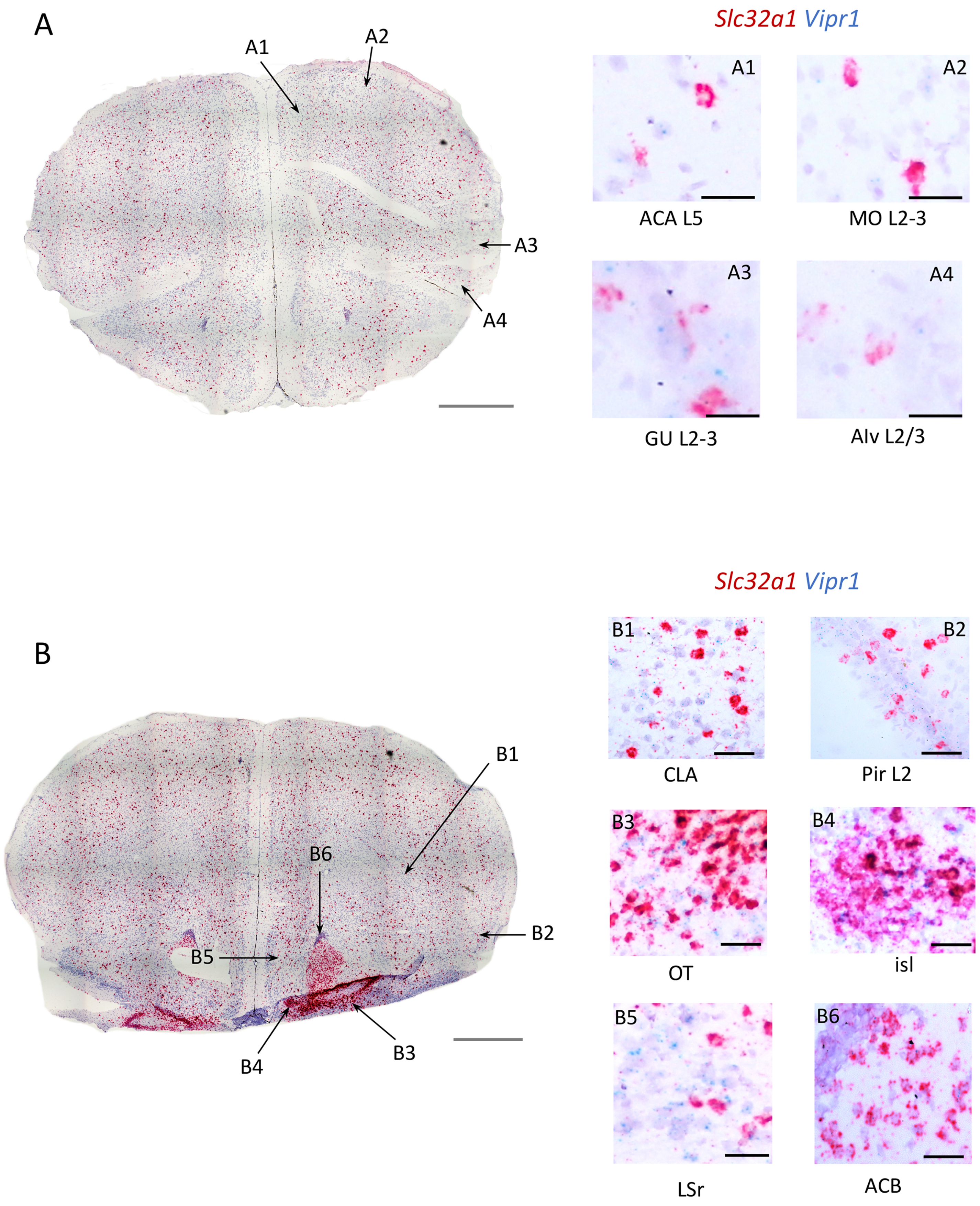
A
A1
A2
A3
A4
Slc32a1 Vipr1
ACA L5
MO L2-3
GU L2-3
AIv L2/3
B
B1
B2
B3
B4
B5
B6
Slc32a1 Vipr1
CLA
Pir L2
OT
isl
LSr
ACB

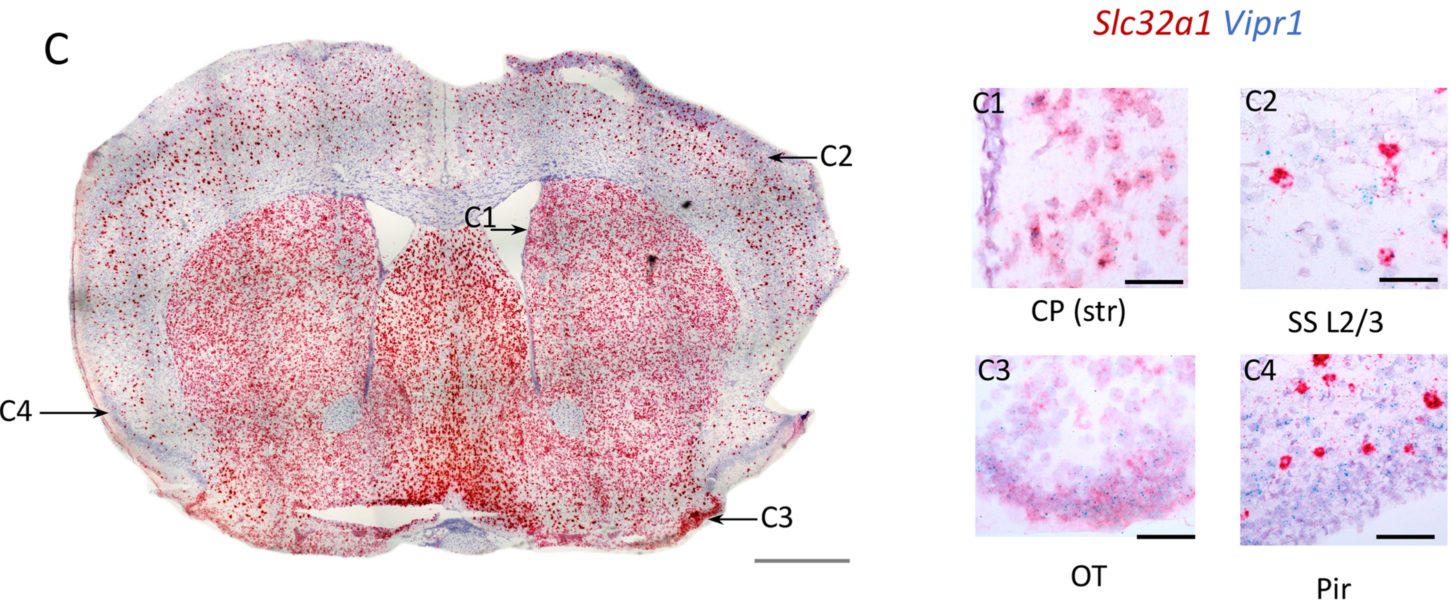
C
Slc32a1 Vipr1
C1
C2
C3
C4
CP (str)
SS L2/3
OT
Pir

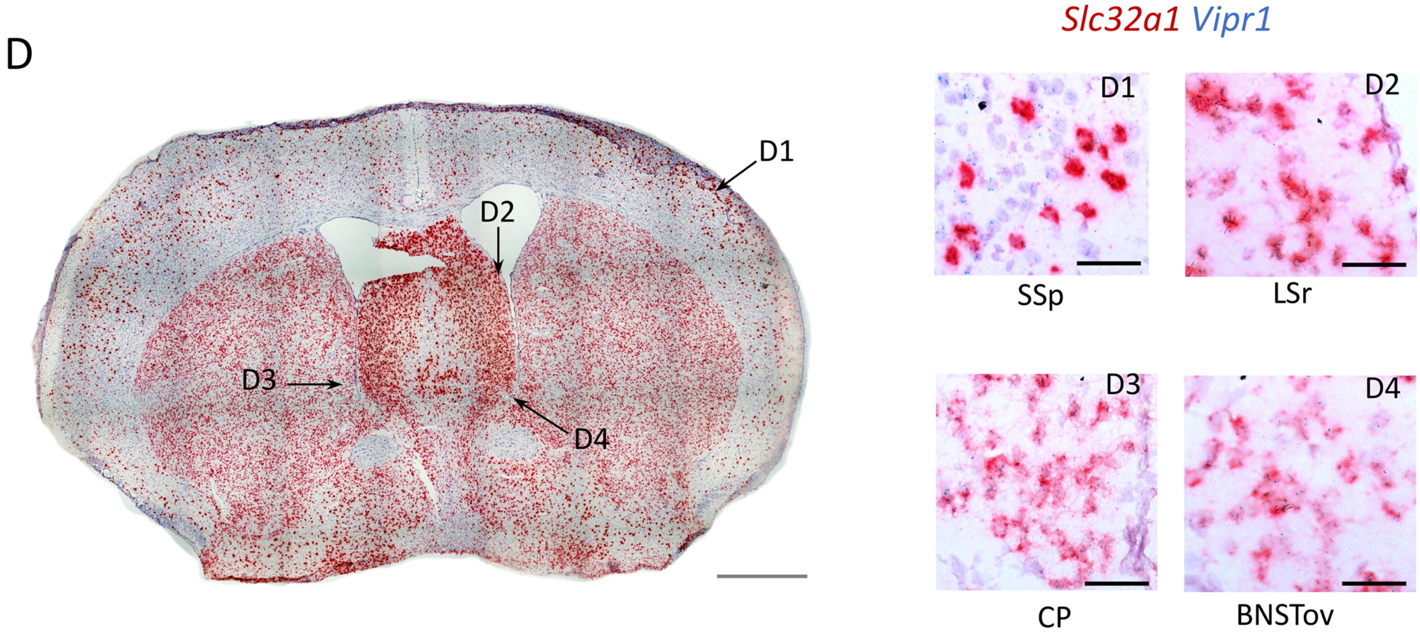
D
Slc32a1 Vipr1
D1
D2
D3
D4
SSp
LSr
CP
BNSTov

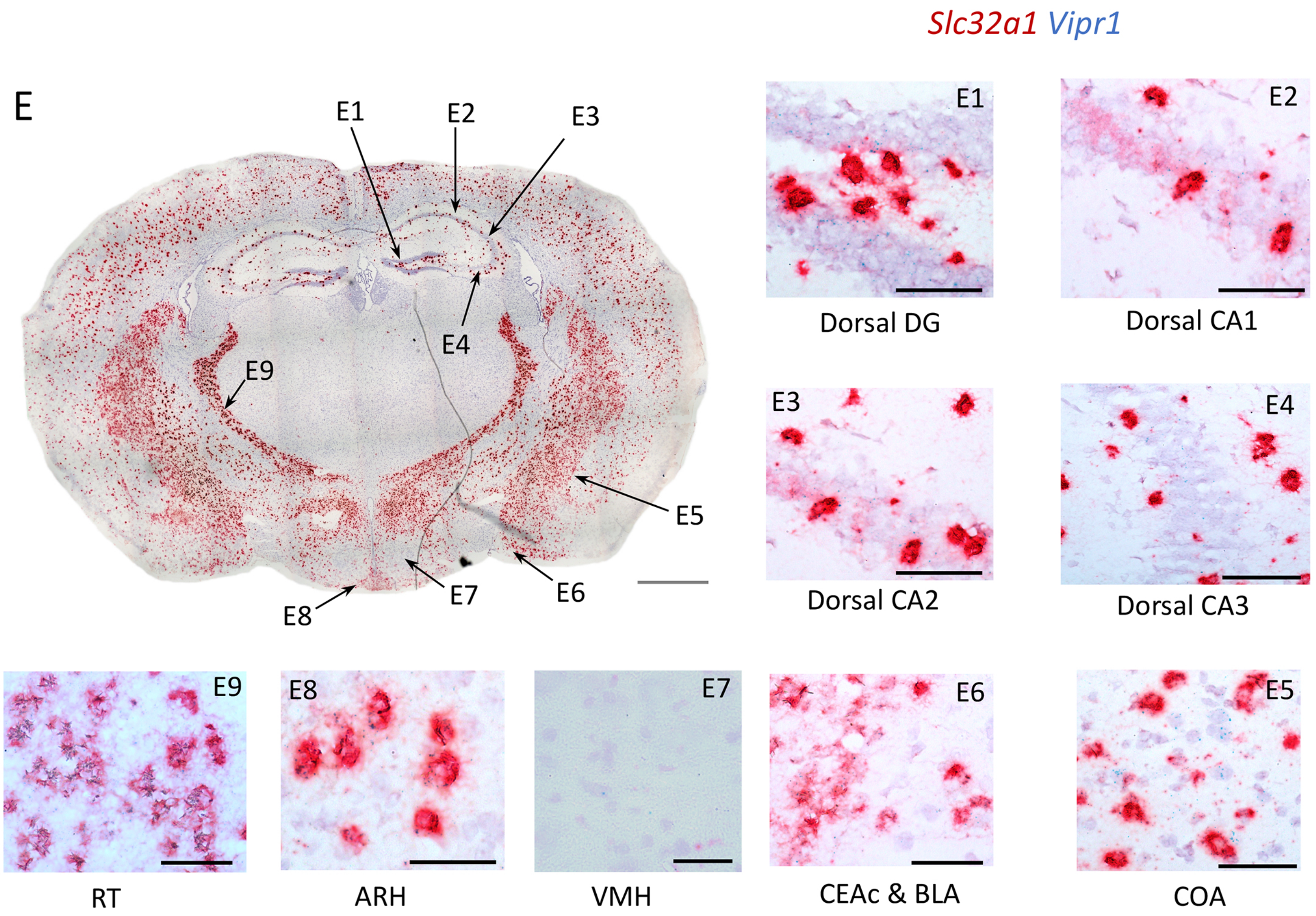
Slc32a1 *Vipr1*
E
E1
E2
E3
E4
E5
E6
E7
E8
E9
Dorsal DG
Dorsal CA1
Dorsal CA2
Dorsal CA3
RT
ARH
VMH
CEAc & BLA
COA

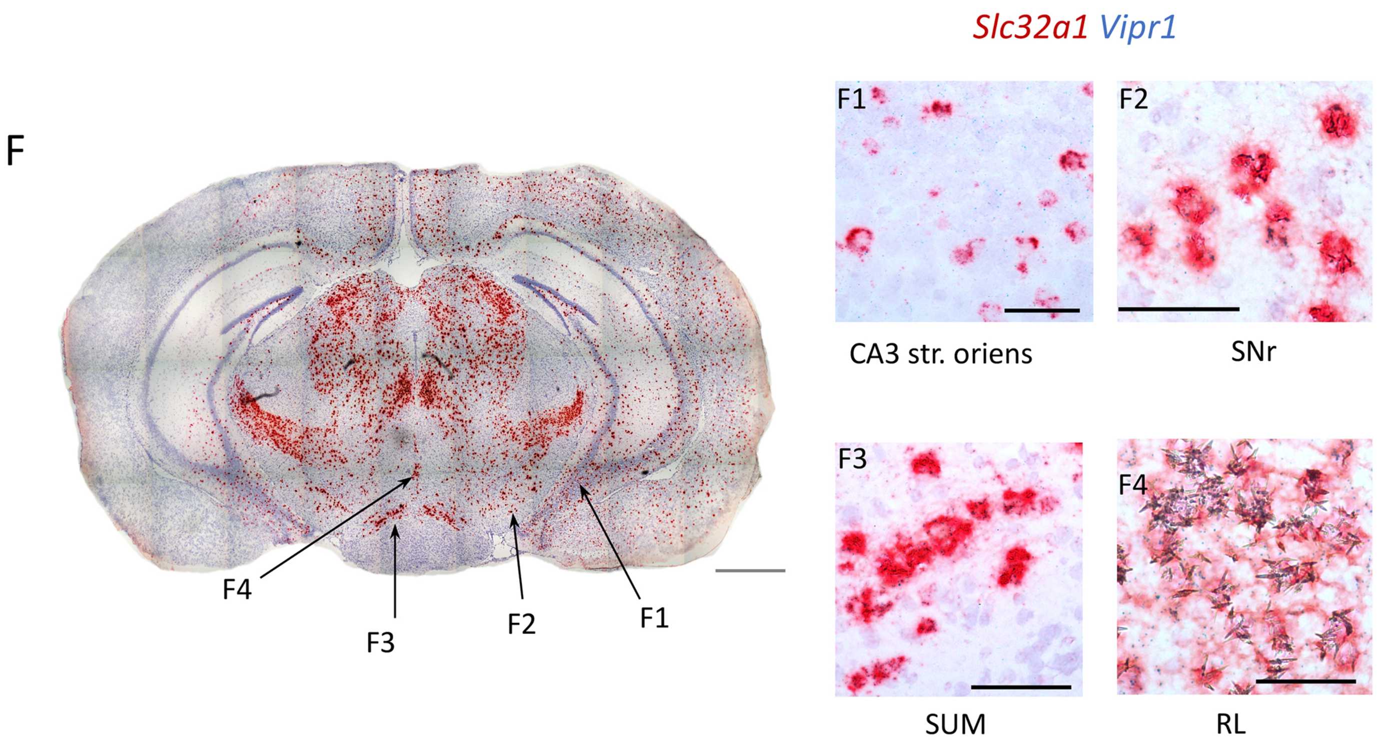
Slc32a1 *Vipr1*
F
F1
F2
F3
F4
CA3 str. oriens
SNr
SUM
RL

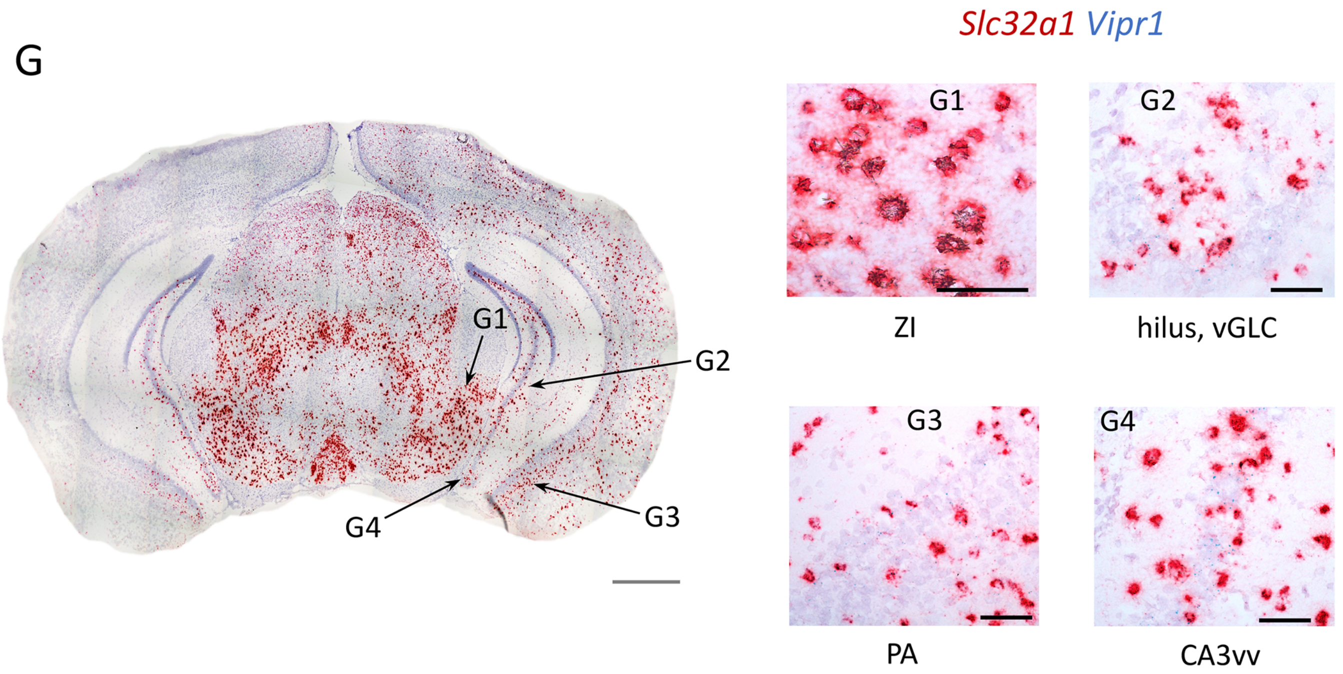
G
Slc32a1 *Vipr1*
G1
G2
G3
G4
ZI
hilus, vGLC
PA
CA3vv

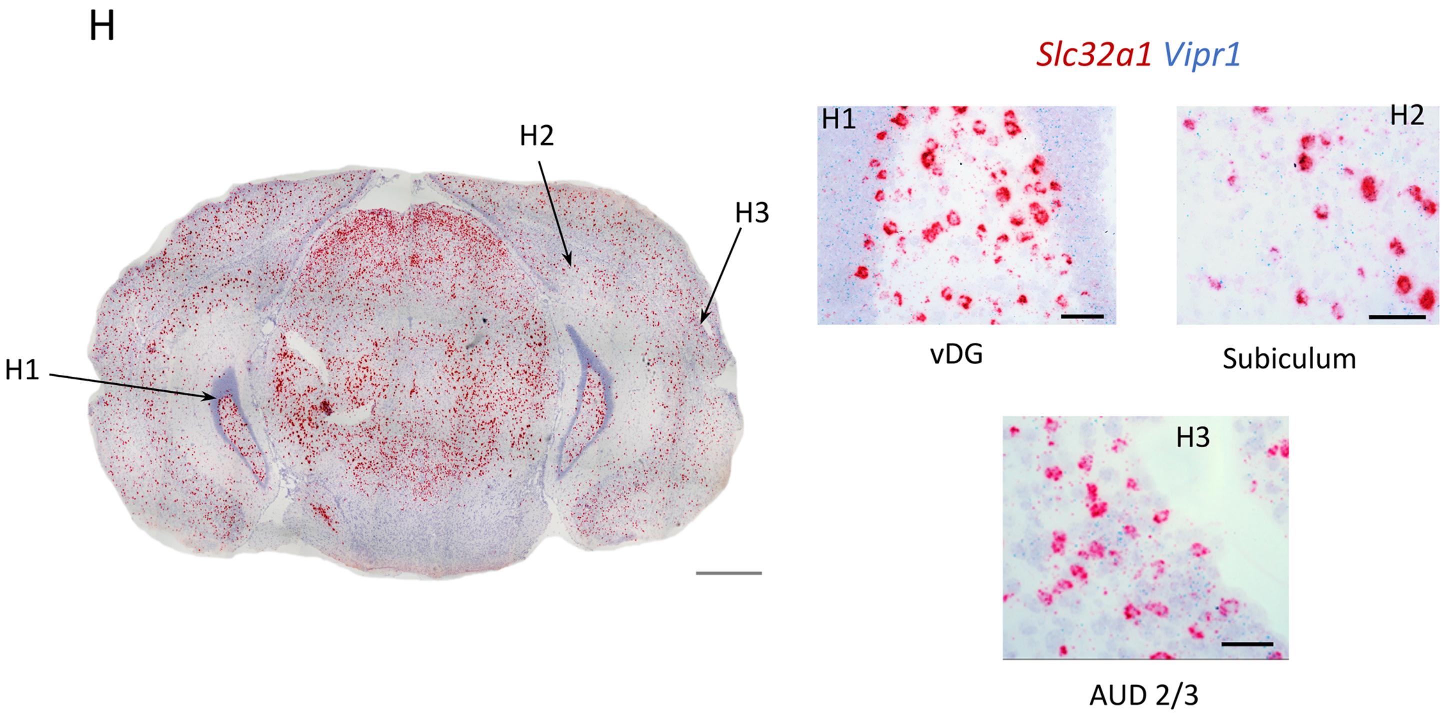
H
Slc32a1 *Vipr1*
H1
H2
H3
vDG
Subiculum
AUD 2/3

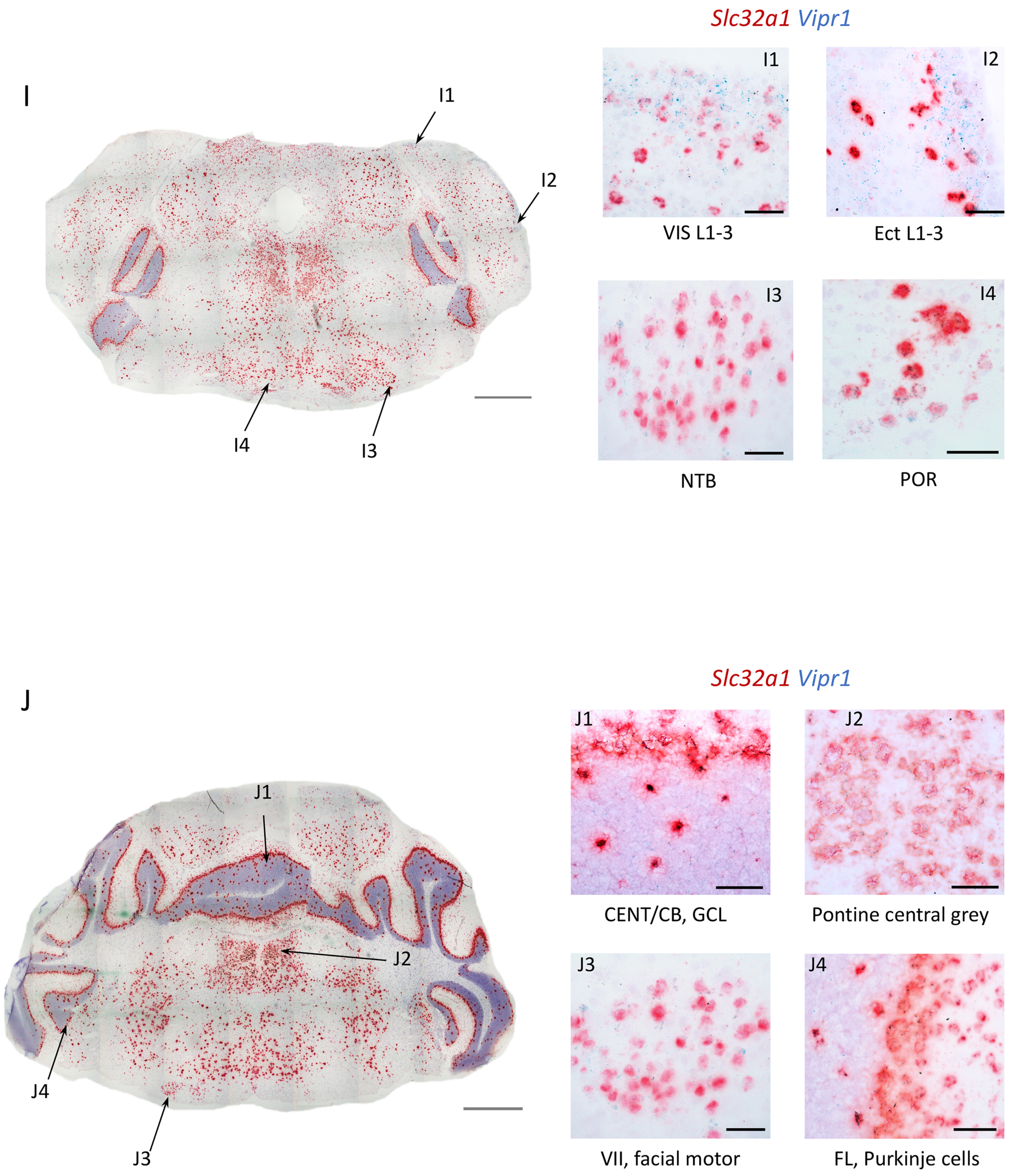
Slc32a1 Vipr1
I
I1
I2
I3
I4
VIS L1-3
Ect L1-3
NTB
POR
Slc32a1 Vipr1
J
J1
J2
J3
J4
CENT/CB, GCL
Pontine central grey
VII, facial motor
FL, Purkinje cells
