# Supplementary material for: Behavioral role of PACAP signaling reflects its selective distribution in glutamatergic and GABAergic neuronal subpopulations
Source: eLife. 2021 Jan 19;10:e61718. doi: 10.7554/eLife.61718 (PMC7875564; doi:10.7554/eLife.61718)
Supplement: Figure 3—source data 2. — Each panel from A to J show a low-magnification image of the coronal section analyzed, indicating with arrows the regions where the corresponding high-magnification photomicrographs were taken. The red signal corresponds to Slc32a1 (the mRNA for VGAT) and the green signal correspond to Vipr2 (the mRNA for VPAC2). The abbreviations correspond to the Allen Brain Map and are indicated in the Figure 3—source data 5, where a comparison with the expression observed in the Vipr2 ISH experiments from Allen (1104 and 1105) and a semiquantitative analysis of the co-expression with VGAT mRNA was done. Scale bar: 2 mm for low-amplification and 50 µm for high-amplification photomicrographs. [file elife-61718-fig3-data2.pdf]

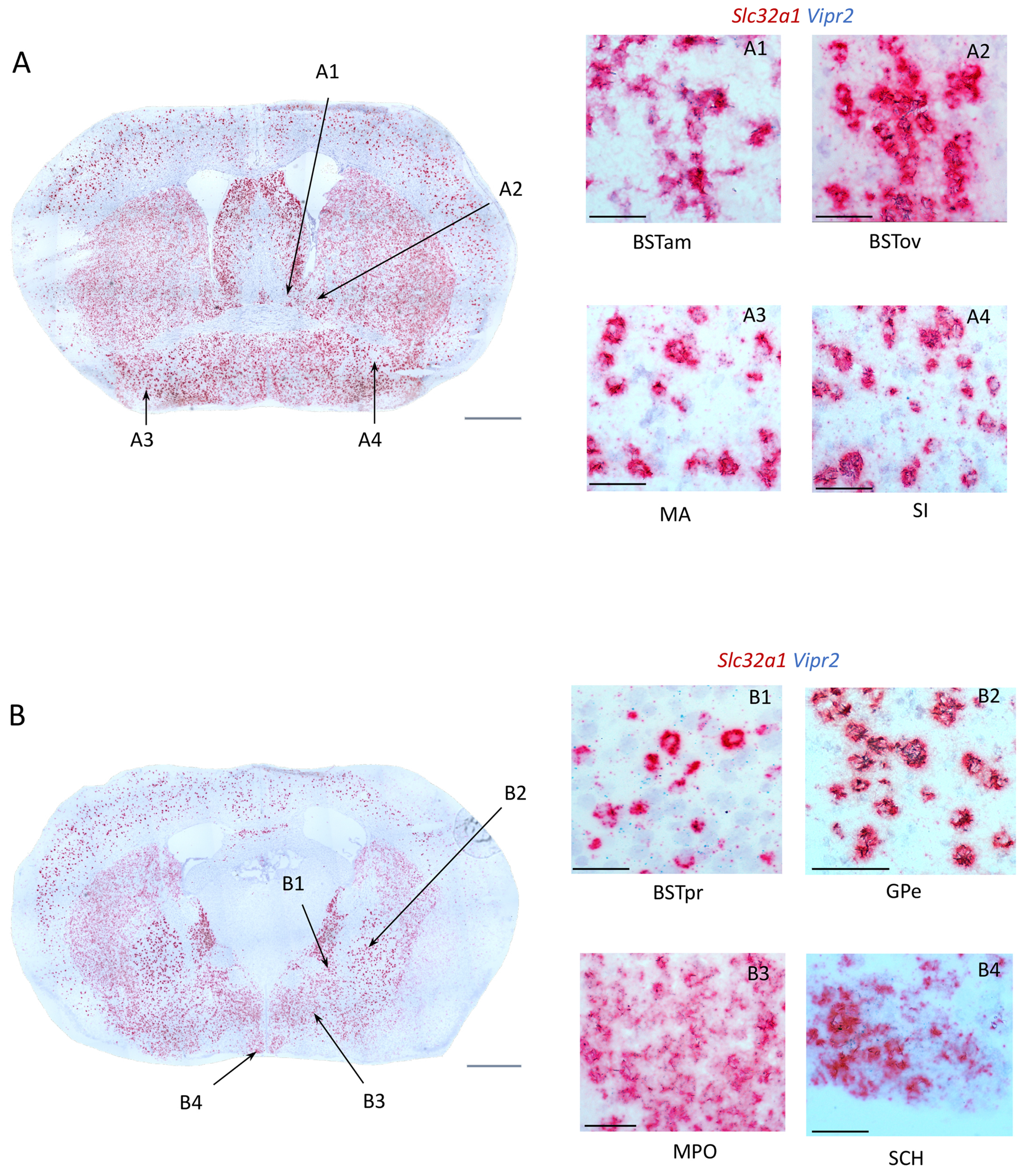
A
A1
A2
A3
A4
Slc32a1 Vipr2
A1
BSTam
A2
BSTov
A3
MA
A4
SI
B
B1
B2
B3
B4
Slc32a1 Vipr2
B1
BSTpr
B2
GPe
B3
MPO
B4
SCH

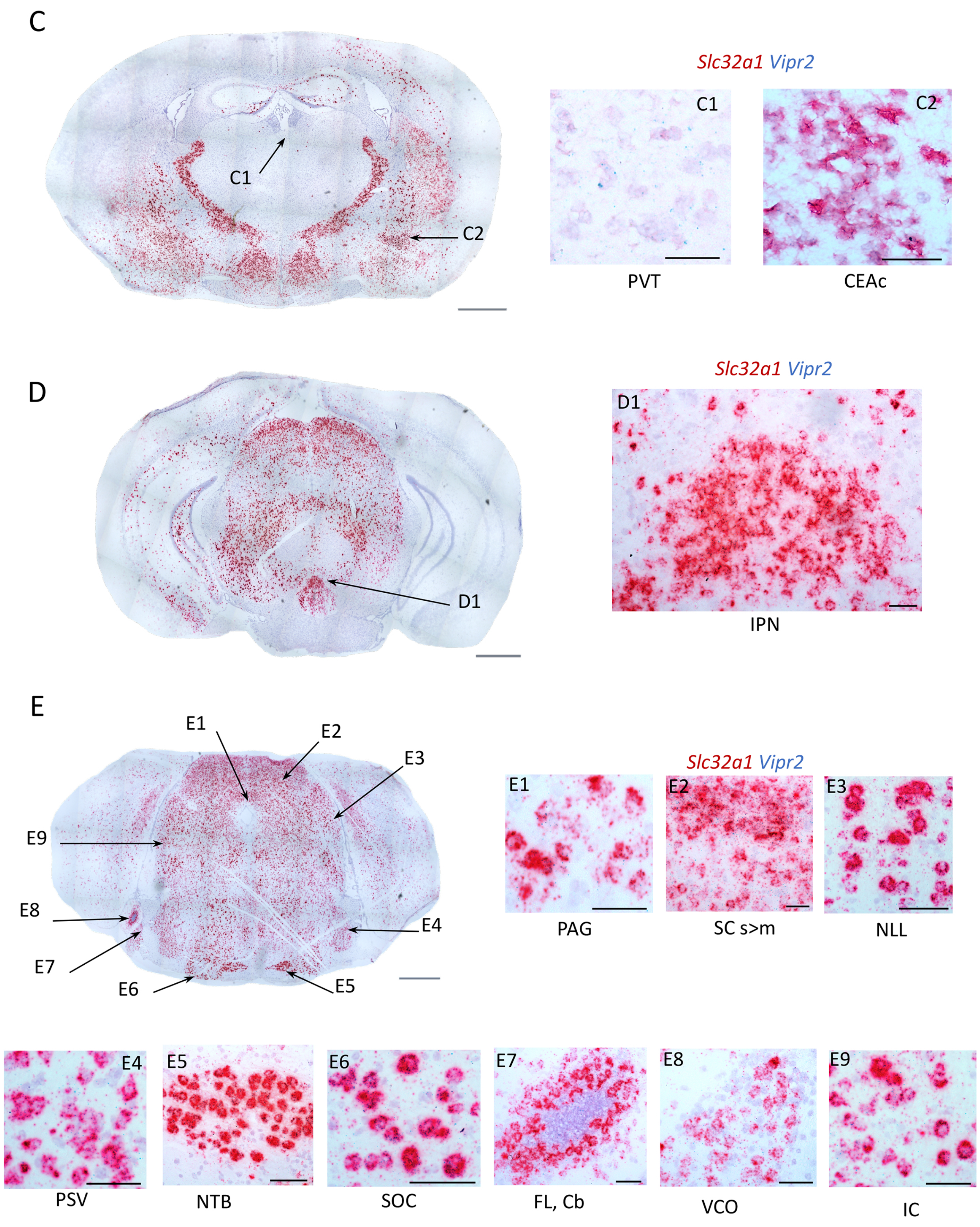
C
C1
C2
Slc32a1 Vipr2
C1
C2
PVT
CEAc
D
D1
Slc32a1 Vipr2
D1
IPN
E
E1
E2
E3
E9
E8
E4
E7
E6
E5
Slc32a1 Vipr2
E1
E2
E3
PAG
SC s>m
NLL
E4
E5
E6
E7
E8
E9
PSV
NTB
SOC
FL, Cb
VCO
IC

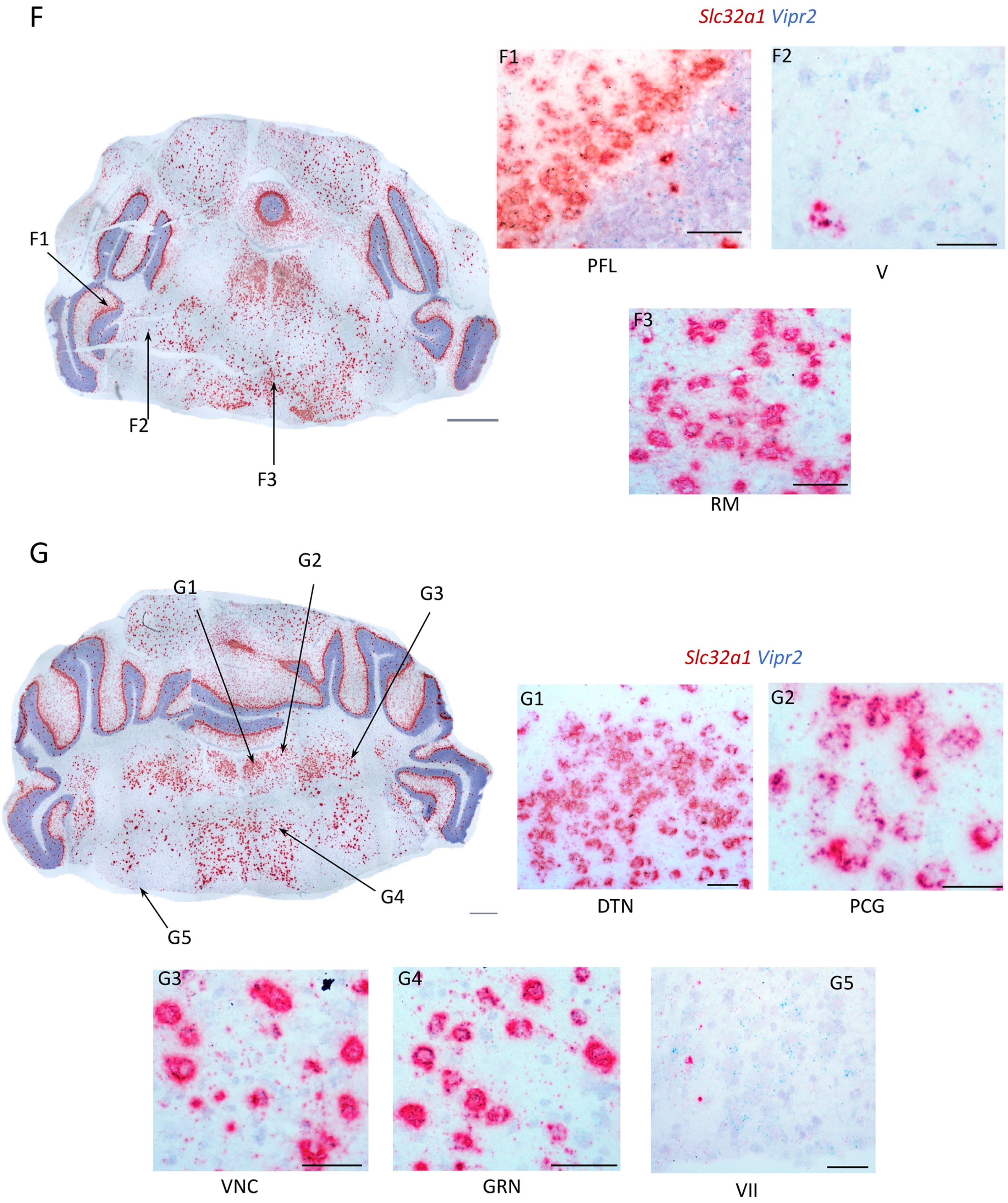
F
Slc32a1 Vipr2
F1
F2
F3
PFL
V
RM
G
G1
G2
G3
G4
G5
Slc32a1 Vipr2
DTN
PCG
VNC
GRN
VII
