# Supplementary material for: Behavioral role of PACAP signaling reflects its selective distribution in glutamatergic and GABAergic neuronal subpopulations
Source: eLife. 2021 Jan 19;10:e61718. doi: 10.7554/eLife.61718 (PMC7875564; doi:10.7554/eLife.61718)
Supplement: Figure 3—source data 3. [file elife-61718-fig3-data3.docx]

Figure 3—source data 3

**Density of *Vipr1* expressing cells in the mouse brain: analysis of *Slc32a1* co-expression and comparison with data from Allen Brain Atlas**

| Abbreviation^a^ | Structure^a^ | Density  Allen^b^ | Density RNAscope | % of *Slc32a1*  (+) | % of *Slc32a1*  (-) |
| --- | --- | --- | --- | --- | --- |
| Cortical plate | | | | | |
| Alv | Alveus | n.o. | + | n.o. | + |
| AOB | accessory olfactory bulb | + | n.o. | - | - |
| MOB | main olfactory bulb | + | n.o. | - | - |
| NLOT | nucleus of the lateral olfactory tract | + | n.o. | - | - |
| PRE | presubiculum | + | n.o. | - | - |
| ACAL5 | anterior cingulate area, dorsal part, layer 5 | ++ | + | n.o. | + |
| CA2v | ventral hippocampus, CA2 | ++ | n.o. | - | - |
| CA2d | dorsal hippocampus, CA2 | ++ | + | + | - |
| CA1d | dorsal hppocampus CA1 | ++ | + | + | n.o. |
| CA3d | dorsal hippocampus, CA3 | ++ | + | + | + |
| COAa | cortical amygdala anterior part | ++ | ++ | + | + |
| dDG-sg | Dorsal dentate gyrus, granule cell layer | ++ | ++ | ++ | n.o. |
| DH | dorsal hippocampus proper | ++ | n.o. | - | - |
| ENTm | entorhinal area, medial part | ++ | n.o. | - | - |
| PAA | piriform-amygdalar area | ++ | n.o. | - | - |
| POST | postsubiculum | ++ | n.o. | - | - |
| vHilus | ventral hilus | ++ | ++ | + | + |
| ACAd1 | anterior cingulate area, dorsal layer 1 | +++ | n.o. | - | - |
| AI | agranular insular area | +++ | n.o. | - | - |
| AON | anterior olfactory nucleus | +++ | n.o. | - | - |
| AUD L2/3 | Auditory cortex, layer 2/3 | +++ | +++ | + | ++ |
| CA3v | field CA3, ventral | +++ | +++ | + | ++ |
| CA3vv | hippocampal formation, ventral CA3c | +++ | +++ | + | ++ |
| vDG-sg | Ventral dentate gyrus, granule cell layer | +++ | +++ | + | ++ |
| ECT | ectorhinal area | +++ | ++++ | + | +++ |
| ENTl | entorhinal area, lateral part | +++ | n.o. | - | - |
| GU | gustatory areas | +++ | ++ | + | + |
| ILA | infralimbic area | +++ | n.o. | - | - |
| MO L2/3 | Motor area | +++ | + | n.o. | + |
| ORB | orbital area | +++ | n.o. | - | - |
| PAR | parasubiculum | +++ | n.o. | - | - |
| PL | prelimbic area | +++ | n.o. | - | - |
| RSP | retrosplenial area | +++ | n.o. | - | - |
| SS | somatosensory areas | +++ | ++ | + | + |
| SUB | subiculum | +++ | +++ | + | ++ |
| PIR | piriform area | ++++ | +++ | + | ++ |
| PTLp | posterior parietal association areas | ++++ | n.o. | - | - |
| TR | postpiriform transition area | ++++ | n.o. | - | - |
| TT | taenia tecta | ++++ | n.o. | - | - |
| TTv | taenia tecta ventral | ++++ | n.o. | - | - |
| VIS | visual areas | ++++ | +++ | + | ++ |
| VISC | visceral area | ++++ | n.o. | - | - |
| Cortical subplate | | | | | |
| LA | lateral amygdalar nucleus | ++ | n.o. | - | - |
| BLA | basolateral amygdalar nucleus | +++ | + | + | n.o. |
| BMA | basomedial amygdalar nucleus | +++ | n.o. | - | - |
| CLA | claustrum | +++ | +++ | + | ++ |
| PA | posterior amygdalar nucleus | +++ | ++ | + | + |
| Epd | endopiriform nucleus | ++++ | n.o. | - | - |
| Cerebral nuclei | | | | | |
| BSTov | bed nucleus of stria terminalis, oval nucleus | n.o. | ++ | ++ | n.o. |
| LSr | lateral septal nucleus rostral | n.o. | +++ | + | ++ |
| AAA | anterior amygdalar area | + | n.o. | - | - |
| CEAc | central amygdalar nucleus, | + | + | + | n.o. |
| IA | intercalated amygdalar nucleus | + | n.o. | - | - |
| Isl | Islands of Calleja | + | ++ | n.o. | ++ |
| MEA | medial amydgala, anterodorsal part | + | n.o. | - | - |
| NDB | diagonal band nucleus | + | n.o. | - | - |
| SI | substantia innominata | + | n.o. | - | - |
| ACB | nucleus accubens | ++ | ++ | n.o. | ++ |
| CP | caudoputamen | ++ | +++ | n.o. | +++ |
| FS | fundus of striatum | ++ | n.o. | - | - |
| OT | olfactory tubercle | ++ | ++++ | +++ | + |
| Interbrain | | | | | |
| RT | reticular nucleus of the thalamus | n.o. | + | n.o. | + |
| AD | anterodorsal nucleus of the thalamus | + | n.o. | - | - |
| AV | anteroventral nucleus of the thalamus | + | n.o. | - | - |
| LH | lateral habenula | + | n.o. | - | - |
| PVT | paraventricular nucleus of the thalamus | + | n.o. | - | - |
| Hypothalamus | | | | | |
| ARH | Arcuate hypothalamic nucleus | n.o. | + | + | n.o. |
| SUM | supramammilary nucleus | n.o. | + | + | n.o. |
| VMH | ventromedial hypothalamic nucleus | n.o. | + | n.o. | + |
| ZI | zona incerta | n.o. | + | + | n.o. |
| PH | posterior hypothalamic nucleus | + | n.o. | - | - |
| PM | premammilary nucleus | + | n.o. | - | - |
| SCH | suprachiasmatic nucleus | + | n.o. | - | - |
| SO | supraoptic nucleus | + | n.o. | - | - |
| Midbrain | | | | | |
| SNr | substantia nigra reticulata | - | ++ | ++ | n.o. |
| PBG | parabigeminal nucleus | + | n.o. | - | - |
| RL | rostral linear nucleus raphe | + | +++ | +++ | - |
| Hindbrain | | | | | |
| PCG | pontine central grey | n.o. | ++ | ++ | n.o. |
| POR | Superior olivary complex, periolivary region | n.o. | + | + | n.o. |
| rV | trigeminal reticular nucleus | n.o. | n.o. | - | - |
| LC | locus coeruleus | + | n.o. | - | - |
| PG | pontine grey | + | n.o. | - | - |
| Medulla | | | | | |
| NTB | Nucleus of the trapezoid body | n.o. | ++ | ++ | n.o. |
| VII | Facial motor nucleus | n.o. | ++ | ++ | n.o. |
| NTS | nucleus of the tractus solitarius | + | n.o. | - | - |
| Cerebellum | | | | | |
| CENT | Central lobule of cerebellum | n.o. | + | n.o. | + |
| CBgcl | granular layer of cerebellum | + | n.o. | - | - |
| CBpj | Purkinje cell layer of cerebellum | + | ++++ | ++++ | - |
| FLgr | cerebellar cortex, flocculus | + | + | + | n.o. |

Density is reported in a semiquantitative way depending on the percentage of the Nissl stained nuclei expressing VipR2 mRNA: for 76%-100% (++++), for 51% -75% (+++), for 26% - 50% (++), for 1% 25% (+) and when there was no expression (-). In the cases where it was not possible to observe the region it was indicated as (n.o).

^"a"^ Nomenclature, abbreviatures and functional classification are based on the Allen Mouse Brain Atlas.

^"b"^ Evaluation of the VipR1 mRNA density through all the coronal and sagittal sections of the Allen ISH experiments 73927619 and 77924538. Density was reported in a semiquantitative way depending on the percentage of the Nissl stained nuclei expressing VipR2 mRNA, i.e: (++++) for 76%-100% , (+++) for 51% -75%, (++) for 26% - 50%, (+) for 1% 25%.

"n.o": no signal observed

"-": not applicable
