# Supplementary material for: Behavioral role of PACAP signaling reflects its selective distribution in glutamatergic and GABAergic neuronal subpopulations
Source: eLife. 2021 Jan 19;10:e61718. doi: 10.7554/eLife.61718 (PMC7875564; doi:10.7554/eLife.61718)
Supplement: Figure 3—source data 4. [file elife-61718-fig3-data4.docx]

Figure 3—source data 4

**Density of *Vipr2* expressing cells in the mouse brain: analysis of *Slc32a1* co-expression and comparison with data from Allen Brain Atlas**

| Abbreviation^a^ | Structure name^a^ | Density  Allen^b^ | Density RNAscope | % of *Slc32a1*  (+) | % of  *Slc32a1*  (-) |
| --- | --- | --- | --- | --- | --- |
| Cortical plate | | | | | |
| DG-sg dorsal | dentate gyrus, granule cell layer, dorsal tier | +++ | n.o. | - | - |
| PIR | piriform area | n.o. | n.o. | - | - |
| Cerebral nuclei | | | | | |
| AAA | anterior amygdalar area | - | n.o. | - | - |
| BSTam | bed nucleus of stria terminalis, anterior division, anteromedial area | - | ++ | ++ | n.o. |
| BSTov | bed nucleus of stria terminalis, anterior division, oval nucleus | +++ | +++ | +++ | n.o. |
| BSTpr | bed nucleus of stria terminalis, posterior division principal nucleus | + | +++ | ++ | + |
| CEAc | central amygdalar nucleus, capsular part | +++ | ++ | ++ | n.o. |
| GPe | globus pallidus, external segment | - | + | + | n.o. |
| MA | magnocellular nuclei | - | + | + | n.o. |
| SI | substantia innominata | - | + | + | n.o. |
| Interbrain | | | | | |
| AM | anteromedial nucleus | ++ | n.o. | - | - |
| AVPV | anteroventral periventricular nucleus | ++++ | n.o. | - | - |
| LD | lateral dorsal nucleus of the thalamus | + | + | - | + |
| LGd | dorsal part of the lateral geniculate complex | +++ | + | - | + |
| LGv | ventral part of the lateral geniculate complex | +++ | n.o. | - | - |
| LH | lateral habenula | + | n.o. | - | - |
| LP | lateral posterior nucleus of the thalamus | + | n.o. | - | - |
| MG | medial geniculate complex | +++ | + | - | + |
| PVT | paraventricular nucleus of the thalamus | + | ++ | n.o. | ++ |
| RE | nucleus of reuniens | + | n.o. | - | - |
| RT | reticular nucleus of the thalamus | +++ | n.o. | - | - |
| VAL | ventral anterior-lateral complex of the thalamus | ++ | + | - | + |
| VM | ventral medial nucleus of the thalamus | ++ | + | - | + |
| VPL | ventral posterolateral nucleus of the thalamus | +++ | + | - | + |
| VPM | ventral posteromedial nucleus of the thalamus | +++ | + | - | + |
| Hypothalamus | | | | | |
| LPO | lateral preoptic area | n.o. | +++ | +++ | n.o. |
| MPO | medial preoptic area | n.o. | +++ | +++ | n.o. |
| SCH | suprachiasmatic nucleus | ++++ | +++ | +++ | n.o. |
| VMH | ventromedial hypothalamic nucleus | + | n.o. | - | - |
| Midbrain | | | | | |
| EW | Edinger-Westphal nucleus | ++ | n.o. | - | - |
| IC | inferior colliculus | n.o. | ++ | ++ | n.o. |
| IPN | interpeduncular nucleus | n.o. | +++ | +++ | n.o. |
| PAG | periaqueductal gray | n.o. | ++ | ++ | n.o. |
| SCm | superior colliculus, motor related | n.o. | ++ | ++ | n.o. |
| SCs | superior colliculus, sensory related | n.o. | +++ | +++ | n.o. |
| Hindbrain | | | | | |
| DTN | Dorsal tegmental nucleus | n.o. | +++ | +++ | n.o. |
| PSV | Principal sensory nucleus of trigeminal nerve | n.o. | +++ | ++ | + |
| V | Motor nucleus of the trigeminal | +++ | +++ | + | ++ |
| NLL | nucleus of lateral lemniscus | n.o. | ++ | ++ | n.o. |
| PCG | pontine central grey | n.o. | + | + | n.o. |
| POR | Superior olivary complex, periolivary region | +++ | n.o. | - | - |
| RPO | nucleus raphe pontis | +++ | + | + | - |
| SOC | superior olivary complex | ++++ | ++ | + | n.o. |
| Medulla | | | | | |
| VCN | dorsal cochlear nucleus | n.o. | ++ | + | + |
| GRN | gigantocellular reticular nucleus | + | + | + | n.o. |
| VNC | vestibular nuclei | n.o. | ++ | + | + |
| NTB | Nucleus of the trapezoid body | n.o. | ++++ | ++++ | n.o. |
| RM | nucleus raphe magnus | n.o. | + | + | n.o. |
| VII | Facial motor nucleus | + | +++ | n.o. | +++ |
| Cerebellum | | | | | |
| FLgr | cerebellar cortex, flocculus | + | ++ | + | + |
| PFLgr | Paraflocculus, granule layer | n.o. | +++ | n.o. | +++ |
| PFLmol | Paraflocculus, molecular layer | n.o. | ++ | ++ | n.o. |

Density is reported in a semiquantitative way depending on the percentage of the Nissl stained nuclei expressing VipR2 mRNA: for 76%-100% (++++), for 51% -75% (+++), for 26% - 50% (++), for 1% 25% (+) and when there was no expression (-). In the cases where it was not possible to observe the region it was indicated as (n.o).

^"a"^ Nomenclature, abbreviations and functional classification are based on the Allen Mouse Brain Atlas.

^"b"^ Evaluation of the VipR2 mRNA density through all the coronal and sagittal sections of the Allen ISH experiments 1104 and 1105. Density was reported in a semiquantitative way depending on the percentage of the Nissl stained nuclei expressing VipR2 mRNA, i.e: (++++) for 76%-100% , (+++) for 51% -75%, (++) for 26% - 50%, (+) for 1% 25% and (-) when there was no expression. In the cases where it was not possible to observe the region it was indicated as (n.o).

"n.o": no signal observed

"-": not applicable
