# Supplementary material for: Behavioral role of PACAP signaling reflects its selective distribution in glutamatergic and GABAergic neuronal subpopulations
Source: eLife. 2021 Jan 19;10:e61718. doi: 10.7554/eLife.61718 (PMC7875564; doi:10.7554/eLife.61718)
Supplement: Figure 3—source data 5. [file elife-61718-fig3-data5.docx]

**Figure 3–source data 5**

Density distribution of PAC1 expressing cells in selective cortical regions

| Bregma ≈ 0.14 mm |  | Bregma ≈ 1.7 mm |  |
| --- | --- | --- | --- |
| ACAd / ACAv | **PAC1** | **RSPd / RSPv** | **PAC1** |
| I | ++++ | I | ++++ |
| II/III | +++++ | II/III | +++++ |
| V | +++++ | V | ++++ |
| VI | +++++ | VI | +++ |
| Mop |  | **SSp / SSs** |  |
| I | +++ | I | +++ |
| II/III | +++++ | II/III | +++++ |
| IV | ++++ | IV | ++++ |
| V | ++++ | V | ++++ |
| VI | +++ | VI | +++ |
| SSs / SSp |  | **VISC** |  |
| I | +++ | I | +++ |
| II/III | +++++ | II/III | +++++ |
| IV | ++++ | IV | ++++ |
| V | ++++ | V | ++++ |
| VI | ++++ | VI | ++++ |
| VISC / GU / AI |  | **AIp** |  |
| I | +++ | I | +++ |
| II/III | +++++ | II/III | +++++ |
| IV | ++++ | V | +++ |
| V | ++++ | VI | +++++ |
| VI | +++++ |  |  |

Semiquantitative annotations are used here (the percentage of PAC1 expressing cell/total Nissl stained nuclei: “+++”, moderate (60%-40%); “++++”, intense (80%-60%); “+++++”, very intense (>80%). ACAd: anterior cingulate area dorsal; ACAv: anterior cingulate area ventral; AI: agranular insular area; AIp: agranular insular area, posterior part; GU: gustatory area; Mop: primary motor area; RSPd: retrosplenial area dorsal; RSPv: retrosplenial area ventral; SSp: primary somatosensory area; SSs: supplemental somatosensory area; VISC: visceral area.
