# Supplementary material for: Behavioral role of PACAP signaling reflects its selective distribution in glutamatergic and GABAergic neuronal subpopulations
Source: eLife. 2021 Jan 19;10:e61718. doi: 10.7554/eLife.61718 (PMC7875564; doi:10.7554/eLife.61718)
Supplement: Figure 8—source data 1. [file elife-61718-fig8-data1.docx]

FIGURES 8 SOURCE DATA

Figure 8C: pixel analysis reflecting total distance traveled in each of the four quadrants

| QUADRANT | WILD TYPE | | | | |  |  | PACAP-KO | | | | |  |  |  |
| --- | --- | --- | --- | --- | --- | --- | --- | --- | --- | --- | --- | --- | --- | --- | --- |
|  | 1 | 2 | 3 | 4 | 5 | **AVG** | **SEM** | 1 | 2 | 3 | 4 | 5 | **AVG** | **SEM** | **T.Test WT-KO** |
| 1 | 29200 | 19878 | 17603 | 34274 | 31546 | **26500** | **3288** | 50490 | 38968 | 50912 | 30514 | 47317 | **43640.2** | **3921** | **0.01** |
| 2 | 34553 | 24749 | 29467 | 40083 | 48562 | **35483** | **4150** | 44354 | 42074 | 34833 | 39178 | 50907 | **42269.2** | **2682** | 0.21 |
| 3 | 50242 | 53120 | 46308 | 41079 | 47444 | **47639** | **2021** | 36244 | 40819 | 44258 | 45898 | 47151 | **42874** | **1969** | 0.13 |
| 4 | 39745 | 39719 | 52948 | 40028 | 29828 | **40454** | **3676** | 46856 | 40824 | 40309 | 35804 | 42995 | **41357.6** | **1805** | 0.83 |

Figure 8D: Numbers of freezing, complete sniff>retreat or partial sniff>retreat episodes

|  | **PACAP KO** | | | | | | | | | **WILD TYPE** | | | | | | | | |
| --- | --- | --- | --- | --- | --- | --- | --- | --- | --- | --- | --- | --- | --- | --- | --- | --- | --- | --- |
| Complete retreat | 1 | 1 | 1 | 1 | 1 | 2 | 2 | 2 | 1 | 4 | 6 | 7 | 5 | 2 | 6 | 10 | 4 | 9 |
| Partial retreat | 4 | 3 | 2 | 1 | 2 | 1 | 0 | 3 | 2 | 0 | 0 | 0 | 0 | 0 | 0 | 0 | 0 | 0 |
| Freezing | 3.4 | 6.3 | 2.7 | 2.7 | 2.4 | 0 | - | 3.4 | 4.5 | 6 | 18.7 | 10.7 | 10.9 | - | 17.3 | 9.5 | 25.2 | 16 |

| Multiple T-Tests | Significant? | P value | Mean of KO | Mean of WT | Difference | SE of difference | t ratio | df | Adjusted P Value |
| --- | --- | --- | --- | --- | --- | --- | --- | --- | --- |
| Complete retreat | Yes | <0.0001 | 1.3 | 5.9 | -4.6 | 0.86 | 5.3 | 16 | 0.0002 |
| Partial retreat | Yes | 0.0002 | 2.0 | 0.0 | 2.0 | 0.41 | 4.9 | 16 | 0.0003 |
| Freezing | Yes | 0.0002 | 3.2 | 14 | -11 | 2.3 | 4.9 | 14 | 0.0003 |

Figure 8E: Number of cells expressing fos RNA 45 min after the predator odor test

|  | **WILD TYPE** | | | | | | | **PACAP-KO** | | | | | | |
| --- | --- | --- | --- | --- | --- | --- | --- | --- | --- | --- | --- | --- | --- | --- |
| OB ml | 37 | 54 | 52 | 46 | 36 | 42 | 44 | 22 | 17 | 20 | 18 | 15 | 25 | 19 |
| OB gl | 21 | 18 | 20 | 23 | 25 | 21 | 22 | 8 | 5 | 6 | 9 | 8 | 7 | 8 |
| MeA | 12 | 12 | 11 | 14 | 11 | 11 | 13 | 6 | 6 | 3 | 8 | 5 | 2 | 5 |
| CeA | 9 | 5 | 7 | 7 | 5 | 8 | 6 | 3 | 2 | 3 | 5 | 2 | 3 | 1 |
| MS | 7 | 7 | 6 | 4 | 5 | 6 | 5 | 4 | 3 | 3 | 3 | 4 | 5 | 4 |
| LS | 14 | 13 | 12 | 13 | 14 | 15 | 17 | 5 | 4 | 4 | 8 | 5 | 4 | 6 |
| BST | 11 | 13 | 12 | 12 | 10 | 9 | 13 | 4 | 5 | 3 | 3 | 4 | 2 | 4 |
| VMH | 14 | 12 | 15 | 8 | 10 | 11 | 13 | 5 | 3 | 6 | 4 | 7 | 5 | 4 |
| IL | 18 | 16 | 15 | 16 | 16 | 12 | 17 | 10 | 9 | 13 | 12 | 8 | 9 | 8 |
| PL | 22 | 19 | 17 | 17 | 22 | 23 | 18 | 8 | 12 | 9 | 8 | 15 | 12 | 16 |
| CG | 26 | 25 | 20 | 22 | 25 | 25 | 27 | 16 | 14 | 13 | 11 | 9 | 7 | 11 |
| PB | 42 | 25 | 28 | 39 | 23 | 33 | 25 | 12 | 6 | 7 | 9 | 5 | 8 | 4 |
| MC IV | 6 | 3 | 5 | 2 | 4 | 7 | 4 | 27 | 25 | 20 | 30 | 24 | 32 | 29 |
| MC V | 5 | 6 | 7 | 5 | 7 | 4 | 3 | 40 | 29 | 32 | 36 | 30 | 33 | 35 |

| Multiple T-Tests | Significant? | P value | Mean of WT | Mean of KO | Difference | SE of difference | t ratio | df | Adjusted P Value |
| --- | --- | --- | --- | --- | --- | --- | --- | --- | --- |
| OB ml | Yes | <0.0001 | 44.43 | 19.43 | 25.00 | 2.884 | 8.667 | 12.00 | <0.0001 |
| OB gl | Yes | <0.0001 | 21.43 | 7.286 | 14.14 | 0.9897 | 14.29 | 12.00 | <0.0001 |
| MeA | Yes | <0.0001 | 12.00 | 5.000 | 7.000 | 0.8729 | 8.020 | 12.00 | <0.0001 |
| CeA | Yes | 0.0002 | 6.714 | 2.714 | 4.000 | 0.7377 | 5.422 | 12.00 | 0.0023 |
| MS | Yes | 0.0020 | 5.714 | 3.714 | 2.000 | 0.5084 | 3.934 | 12.00 | 0.0298 |
| LS | Yes | <0.0001 | 14.00 | 5.143 | 8.857 | 0.8289 | 10.69 | 12.00 | <0.0001 |
| BST | Yes | <0.0001 | 11.43 | 3.571 | 7.857 | 0.6801 | 11.55 | 12.00 | <0.0001 |
| VMH | Yes | <0.0001 | 11.86 | 4.857 | 7.000 | 1.043 | 6.710 | 12.00 | 0.0003 |
| IL | Yes | <0.0001 | 15.71 | 9.857 | 5.857 | 1.027 | 5.704 | 12.00 | 0.0015 |
| PL | Yes | 0.0002 | 19.71 | 11.43 | 8.286 | 1.567 | 5.287 | 12.00 | 0.0029 |
| CG | Yes | <0.0001 | 24.29 | 11.57 | 12.71 | 1.473 | 8.631 | 12.00 | <0.0001 |
| PB | Yes | <0.0001 | 30.71 | 7.286 | 23.43 | 2.995 | 7.821 | 12.00 | <0.0001 |
| MC IV | Yes | <0.0001 | 4.429 | 26.71 | -22.29 | 1.670 | 13.34 | 12.00 | <0.0001 |
| MC V | Yes | <0.0001 | 5.286 | 33.57 | -28.29 | 1.536 | 18.41 | 12.00 | <0.0001 |
