# Supplementary material for: Behavioral role of PACAP signaling reflects its selective distribution in glutamatergic and GABAergic neuronal subpopulations
Source: eLife. 2021 Jan 19;10:e61718. doi: 10.7554/eLife.61718 (PMC7875564; doi:10.7554/eLife.61718)
Supplement: Figure 9—source data 1. [file elife-61718-fig9-data1.docx]

FIGURE 9: SOURCE DATA

Figure 9E: Number of cells expressing *Slc32a1*, *Slc17a7* and *Slc17a6* in a 0.0314 mm2 area

|  | WT | | | | | | KO | | | | | |
| --- | --- | --- | --- | --- | --- | --- | --- | --- | --- | --- | --- | --- |
| Septum (*Slc32a1*) | 39 | 40 | 37 | 44 | 42 | 36 | 10 | 7 | 10 | 9 | 8 | 8 |
| BST (*Slc32a1*) | 27 | 22 | 28 | 20 | 25 | 21 | 5 | 7 | 4 | 6 | 5 | 4 |
| Cerebelum (*Slc32a1*) | 20 | 24 | 22 | 17 | 25 | 23 | 1 | 3 | 1 | 2 | 3 | 1 |
| Ret. thal. (*Slc32a1*) | 12 | 9 | 7 | 13 | 10 | 11 | 10 | 7 | 8 | 11 | 9 | 10 |
| ACA (*Slc17a7*) | 39 | 40 | 37 | 44 | 42 | 36 | 16 | 14 | 17 | 13 | 20 | 14 |
| STN (*Slc17a6*) | 28 | 32 | 20 | 25 | 29 | 23 | 8 | 3 | 5 | 4 | 5 | 3 |

| Multiple T-Tests | Significant? | P value | Mean of WT | Mean of KO | Difference | SE of difference | t ratio | df | Adjusted P Value |
| --- | --- | --- | --- | --- | --- | --- | --- | --- | --- |
| Septum | Yes | <0.0001 | 39.67 | 8.667 | 31.00 | 1.325 | 23.40 | 10.00 | <0.0001 |
| BST | Yes | <0.0001 | 23.83 | 5.167 | 18.67 | 1.434 | 13.02 | 10.00 | <0.0001 |
| Cerebelum | Yes | <0.0001 | 21.83 | 1.833 | 20.00 | 1.261 | 15.87 | 10.00 | <0.0001 |
| Ret. thal. | No | 0.2999 | 10.33 | 9.167 | 1.167 | 1.067 | 1.093 | 10.00 | 0.2999 |
| ACA | Yes | <0.0001 | 39.67 | 15.67 | 24.00 | 1.619 | 14.82 | 10.00 | <0.0001 |
| STN | Yes | <0.0001 | 26.17 | 4.667 | 21.50 | 1.934 | 11.12 | 10.00 | <0.0001 |
